# Targeting Mechano-Transcription Process as Therapeutic Intervention in Gastrointestinal Disorders

**DOI:** 10.3389/fphar.2021.809350

**Published:** 2021-12-21

**Authors:** Ramasatyaveni Geesala, You-Min Lin, Ke Zhang, Xuan-Zheng Shi

**Affiliations:** Department of Internal Medicine, The University of Texas Medical Branch, Galveston, TX, United States

**Keywords:** bowel obstruction, Crohn’s disease, functional bowel disorders, mechanical stress, gut motility, intestinal fibrosis, visceral pain, gene expreesion

## Abstract

Mechano-transcription is a process whereby mechanical stress alters gene expression. The gastrointestinal (GI) tract is composed of a series of hollow organs, often encountered by transient or persistent mechanical stress. Recent studies have revealed that persistent mechanical stress is present in obstructive, functional, and inflammatory disorders and alters gene transcription in these conditions. Mechano-transcription of inflammatory molecules, pain mediators, pro-fibrotic and growth factors has been shown to play a key role in the development of motility dysfunction, visceral hypersensitivity, inflammation, and fibrosis in the gut. In particular, mechanical stress-induced cyclooxygenase-2 (COX-2) and certain pro-inflammatory mediators in gut smooth muscle cells are responsible for motility dysfunction and inflammatory process. Mechano-transcription of pain mediators such as nerve growth factor (NGF) and brain-derived neurotrophic factor (BDNF) may lead to visceral hypersensitivity. Emerging evidence suggests that mechanical stress in the gut also leads to up-regulation of certain proliferative and pro-fibrotic mediators such as connective tissue growth factor (CTGF) and osteopontin (OPN), which may contribute to fibrostenotic Crohn’s disease. In this review, we will discuss the pathophysiological significance of mechanical stress-induced expression of pro-inflammatory molecules, pain mediators, pro-fibrotic and growth factors in obstructive, inflammatory, and functional bowel disorders. We will also evaluate potential therapeutic targets of mechano-transcription process for the management of these disorders.

## Introduction

**FIGURE 1:**
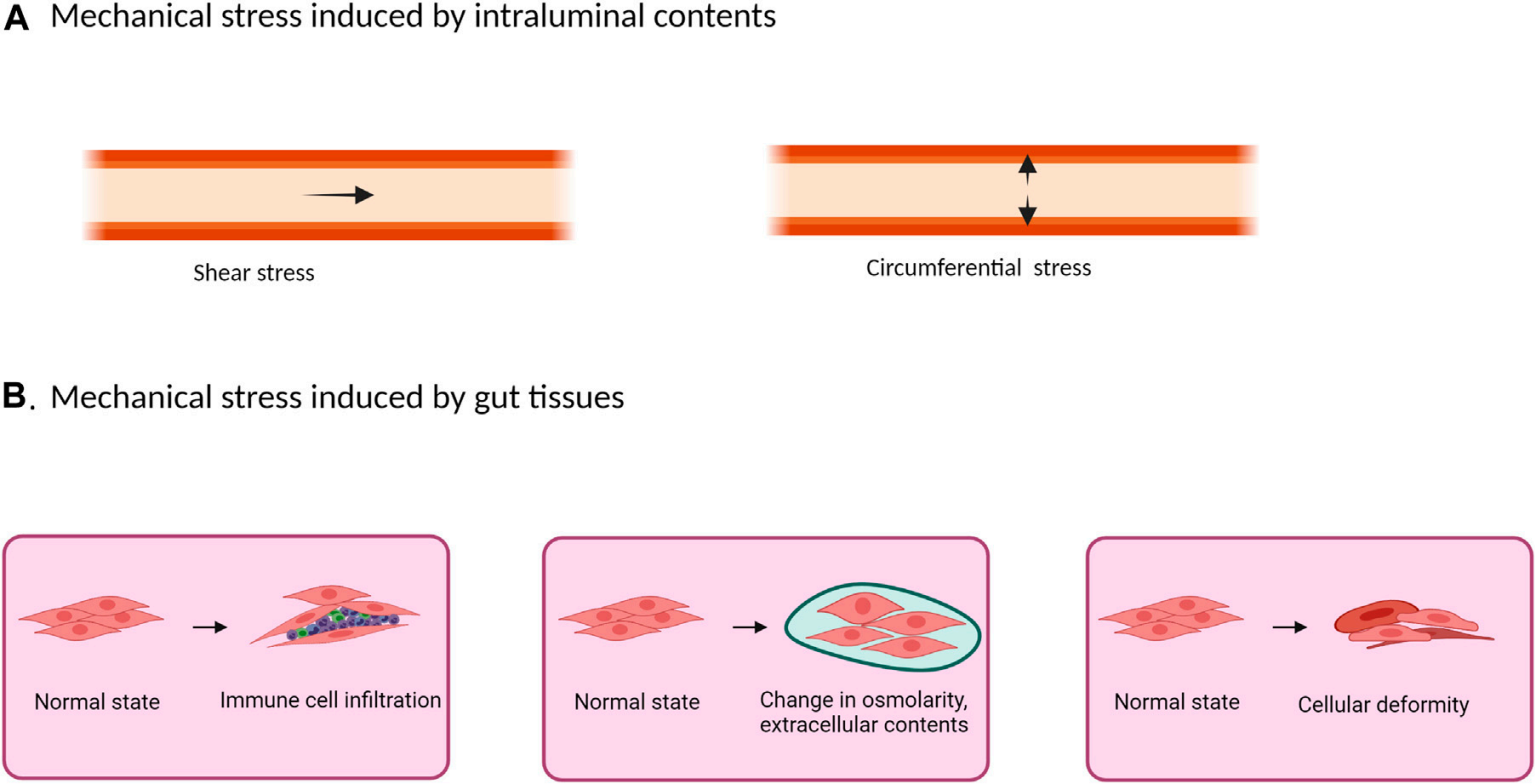
Mechanical stress in the gut may originate from the intraluminal contents **(A)** or from inside the tissues **(B)**. **(A)** The presence and movement of intraluminal contents create shear stress and intraluminal pressure. While shear stress is a transient force generated at the mucosa surface tangential to the GI tract, intraluminal pressure creates a circumferential mechanical stretch perpendicular to the gut wall. **(B)** Physical stretch and compression also exist inside the gastrointestinal tissues in pathological conditions such as in inflammation with inflammatory cell infiltrations, edema, extracellular matrix deposition, and deformation.

The gastrointestinal (GI) tract is subject to mechanical stimuli constantly (Shi, 2017; Sarna and Shi, 2006). Contracting forces generated by gut smooth muscle mechanically digest foods in the lumen and propel the intraluminal contents down through the GI tract (Shi and Sarna, 2005; Murthy, 2006; Karichely and Farrugia, 2007). The intraluminal contents (foods, gas, and fluids) and the movement of the contents present mechanical stress on the gut wall: i.e., pressure and shear stress (Shi, 2017). Shear stress is transient and generated at the mucosa surface tangential to the GI tract. Along with that, intraluminal pressure generates a circumferential mechanical stretch which is perpendicular to the gut wall (Figure 1A). Shear stress primarily affects mucosa and submucosa layers, and its impact on epithelial and enterochromaffin cells has been previously studied *in vitro* (Gayer and Basson, 2009; Linan-Rico et al., 2016; Beyder, 2019). Circumferential stretch, however, affects the whole gut wall including the muscularis externa, and is most remarkable in pathological conditions such as obstructive, inflammatory, and functional bowel disorders. Pathophysiological significance of mechanical stretch in these gastrointestinal disorders have not been reviewed comprehensively.

Under homeostasis, the intraluminal pressure in the gut is about 0 cm H_2_O (Silen, 2005). Up on blockade of intraluminal contents, as in bowel obstruction, it increases up to 8–10 cm H_2_O in the proximal segment of obstruction (Shikata et al., 1983; Silen, 2005). The intraluminal pressure may reach up to 30 to 60 cm H_2_O during peristalsis in the obstructed segment (Shikata et al., 1983; Silen, 2005). In obstruction, overload of luminal contents (food contents, fluids and gas) at proximal segment leads to lumen distention. According to Laplace’s law, the circumferential mechanical stress on the bowel wall, i.e., product of pressure and radius, will be significantly enhanced during obstruction (Russell and Welch, 1990; Shi, 2017). Many GI conditions, i.e., obstructive bowel disorders (OBD), are characterized with lumen distention, and hence mechanical stress (Shi et al., 2011; Lin et al., 2012a). OBDs are a major health concern in both adults and children (Russell and Welch, 1990; Summers, 1999; Silen, 2005). Distension of gut lumen in OBD might be due to either mechanical or functional obstruction (Russell and Welch, 1990; Summers, 1999; Silen, 2005). Functional obstruction is due to neuromuscular dysfunction, such as in achalasia, pyloric stenosis, ileus, Hirschsprung’s disease, and intestinal pseudo-obstruction (Russell and Welch, 1990; Summers, 1999; Nunez et al., 2009; De Giorgio et al., 2011), whereas mechanical obstruction results from extrinsic factors such as adhesions and hernias, or intrinsic factors such as carcinoma and diverticulitis (Shi, 2017). Although circumferential mechanical stretch is most prominent in obstructive bowel disorders, it is also well noticed in functional bowel disorders and inflammatory conditions, i.e., fecal retention, constipation, and stenotic Crohn’s disease (Heredia et al., 2012; Raahave, 2015; Lin et al., 2021b).

Mechanical stress on gut tissues may not only be generated from intraluminal contents as described above, but also from gut tissues themselves (Figure 1B). In gut inflammation, for example, physical stretch and compression exist in the GI tissues, where inflammatory cell infiltrations, tissue deformation, edema, and extracellular matrix deposition (fibrosis) are all considered as mechanical stress (Cox et al., 2008; Gayer and Basson, 2009; Johnson et al., 2013; Levy Nogueira et al., 2015). Furthermore, these changes at the inflammation site may cause partial obstruction (inflammatory stenosis), leading to lumen distention in the bowel segment prior to the site of inflammation (circumferential mechanical stretch) (Katsanos et al., 2010; Shi, 2017). These changes are well represented in transmural inflammation especially stenotic Crohn’s disease, where stenosis is caused not only by inflammation, but also by fibrosis-associated stricture formation (Katsanos et al., 2010; Rieder et al., 2013).

Functional and morphological changes such as dysmotility (Summers et al., 1983; Prihoda et al., 1984), visceral pain (Huang et al., 2005; Lin et al., 2017), muscular hypertrophy and hyperplasia (Gabella, 1975; Gabella, 1990), damages in the enteric nervous system and interstitial cell of Cajal (Chang et al., 2001a; Wedel et al., 2002) have been observed due to excessive mechanical stress. Interestingly, the effect of mechanical stress is very different in different tissues and cells in the gut. For example, mechanical stretch in a rat model of bowel obstruction leads to injury and disruption in enteric nervous system and interstitial cell of Cajal network 7 days after induction of obstruction (Wu et al., 2013; Lin et al., 2014a). The similar stress conditions lead to hyperplasia of gut smooth muscle cells associated with mechanical stress-altered gene transcription, a process referred as “mechano-transcription” (Shi et al., 2011; Li et al., 2012a). Nevertheless, mechanisms underlying mechanical stress-associated dysfunctions and gene expression are not well understood.

To address the pathophysiological mechanisms, prior studies were focused on the dysfunctions several weeks after the initiation of bowel obstruction, a prototype of *in vivo* mechanical stretch (Gabella, 1975; Gabella, 1990; Chang et al., 2001b; Wedel et al., 2002; Bertoni et al., 2004; Won et al., 2006). Although this strategy may be able to document dramatic changes after obstruction, it might have ruled out some possible early cellular and molecular events, that could have led to the morphological and functional changes recorded later (Shi, 2017). Over the last decade, several investigators along with us have tested a new theory to understand bowel dysfunctions in response to mechanical stress. We hypothesized that mechanical stress regulates expression of mechanosensitive genes in the gut, and in the long run, the mechanical stress-altered gene expression may account for bowel dysfunctions (Shi et al., 2011; Li, et al., 2012a; Wehner et al., 2010). Recent studies found that gut smooth muscle cells are highly sensitive to mechanical stress and contribute greatly to mechano-transcription in the GI tract (Shi et al., 2011; Shi, 2017). The cellular process transducing mechanical signals to the altered gene transcription involves mechanosensors (e.g., integrins and stretch-activated ion channels) in the plasma membrane and complex intracellular signaling pathways involving protein kinases C and D, and mitogen-activated protein kinases (MAPKs) (Li et al., 2012a; Li et al., 2012b). Mounting evidence now suggests that mechano-transcription in the GI tract plays a crucial role in motility dysfunction, visceral hypersensitivity, inflammation, and fibrosis in obstructive, inflammatory, and functional disorders.

In this review, we will discuss the effects of mechanical stress in the gut wall. Major focus will be on the process of mechano-transcription, its signal transduction mechanisms and pathophysiological significance in the gut. Furthermore, we will highlight the potential therapeutic targets of the mechano-transcription process for the treatment of obstructive, inflammatory, and functional disorders.

## Mechano-Transcription in the Gastrointestinal Tract

To gain insights into the mechanisms and significance of mechano-transcription in the gut, several *in vitro* and *in vivo* stretch models have been developed (Shi, 2017). Among these, the Flexcell system is possibly the best-established *in vitro* model to study mechanical stretch in cultured cells (Gayer and Basson, 2009; Wehner et al., 2010; Shi et al., 2011; Li et al., 2015). This system uses a computer-regulated bioreactor to apply finely controlled multi-axial static or cyclic strains through vacuum pressure to cells cultured on flexible membrane plates. It has been used to study mechano-transcription *in vitro* in gut smooth muscle cells (SMC) (Wehner et al., 2010; Shi et al., 2011), epithelial cells (Gayer and Basson, 2009), and macrophages (Wehner et al., 2010; Docsa et al., 2020). Another *in vitro* system applies hydrostatic pressure to stretch cells (e.g., intestinal SMC) cultured on silicon membranes (Gutierrez and Perr, 1999). The amount of distension is controlled by volume of fluid added to the chamber beneath the silicon membrane.

To study mechano-transcription *in vivo* in the GI tract, mechanical stretch (i.e., lumen distention) can be established by an intraluminal balloon to apply a desired pressure in the bowel (Lin et al., 2015) or surgically with an obstruction band around the studied area (esophagus, stomach, small intestine or colon) (Shi et al., 2011; Lin et al., 2012b). As mechanical bowel obstruction (BO) is the prototype obstructive disorder in the GI tract, our lab has used extensively the model of partial colon obstruction to understand mechanical regulation of gene expression and bowel functions *in vivo* (Shi et al., 2011; Li et al., 2012a; Lin et al., 2012a; Lin et al., 2017). To induce partial colon obstruction in rodents, a 3-mm wide medical grade silicon band is placed around the distal colon at about 3 cm proximal to the anus. The length of the band (20–21 mm for rat; 10 mm for mice) is 1–2 mm longer than the outer circumference of the colon when the colon segment is filled with a fecal pellet, allowing a partial rather than complete obstruction (Shi et al., 2011). This procedure creates a significant colon distention in the segment proximal to obstruction band, and the external circumference is increased from 20 mm in control to 32–34 mm in obstruction in rats (Li et al., 2012a).

Our lab applied the *in vivo* model of partial colon obstruction to examine the scope of the effect of mechanical stress on gene expression in gut SMC utilizing a comprehensive Affymetrix cDNA array analysis (Shi et al., 2011; Shi, 2017). This study revealed that mechanical stretch dramatically altered the gene expression profile in the distended bowel segment oral to the obstruction site, compared to sham control or the non-stretch aboral segment. There is an at least 2-fold increase in the expression of 309 genes and decrease in the expression of 282 genes in stretched tissues (proximal to obstruction band), comparing to the non-stretch tissues (distal to obstruction). Gene expression of certain pro-inflammatory molecules, pain mediators, growth factors, extracellular matrix proteins, cell signaling molecules is altered by mechanical stretch. (Shi et al., 2011; Shi, 2017). This work demonstrates that mechanical stress substantially alters the gene expression profile in the gut. This altered gene expression may be one of the key mechanisms of bowel dysfunctions in conditions with lumen distention.

### Mechano-Transcription of Prostaglandin-Endoperoxide Synthase-2 (COX-2) in Gut Smooth Muscle Cells

**FIGURE 2:**
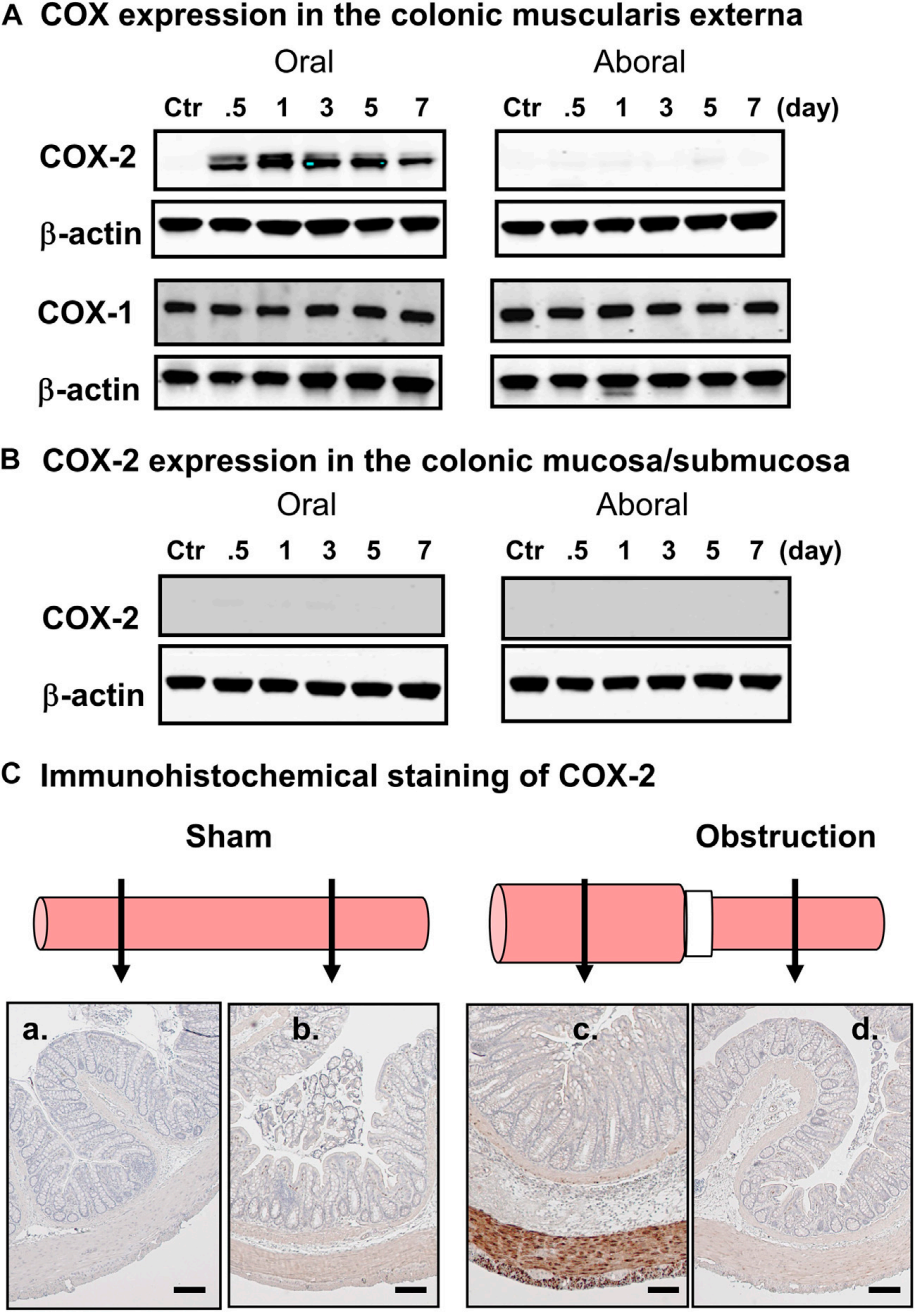
Mechanical stress-induced up-regulation of COX-2 selectively in gut smooth muscle cells. **(A)** Western blot detected up-regulation of COX-2, but not COX-1, in the colonic muscularis externa in the oral segment, but not aboral segment, in relation to the obstruction band. **(B)** Western blot did not detect COX-2 expression in the mucosa/submucosa layer in segments either oral or aboral to the obstruction band. **(C)** Immunohistochemical study of COX-2 expression in segments oral (a, c) and aboral (b, d) colon segments in a sham operated rat (a, b) and a rat with obstruction (c, d) for 3 days. The expression of COX-2 (stained in brown) is detected only in smooth muscle cells in the distended oral segment (c). The results shown here are representative of four independent experiments. Calibration bars represent 50 μm. Some parts of the figure are adapted with permission from Figure 1 of Shi (2017).

One of the upregulated genes detected in the micro-array study of the mechanically distended bowel is prostaglandin-endoperoxide synthase-2 (PTGS-2), also known as cyclooxygenase-2 (COX-2), which has shown a 19-fold increase in expression in the distended colon of BO rats, compared to the non-distended colonic segment aboral to obstruction. This enzyme catalyzes the major rate-limiting step in the synthesis of prostaglandins (PGs), which have profound impacts on gut inflammation, cell proliferation, motility, and visceral pain in the GI tract (Fornai et al., 2005; Krause and DuBois, 2000; Mohajer and Ma, 2000). While COX-1 is expressed constitutively in most of cell types, COX-2 is the inducible form (Krause and DuBois 2000; Mohajer and Ma, 2000). Our corroborative studies found that expression of COX-2 is increased significantly in the muscularis externa of stretched colon segment oral to obstruction, whereas its expression is not changed in the non-stretched segment aboral to obstruction (Figure 2A) (Shi et al., 2011). The expression of COX-1 is not altered either in the oral or aboral segment. Furthermore, immunohistochemical studies confirmed that there is an enhanced COX-2 expression in the smooth muscle cells (circular and longitudinal), but not in mucosa/submucosa, or myenteric plexus (Figures 2B,C). These studies depict that mechano-transcription of COX-2 in bowel obstruction is a phenomenon specific to smooth muscle cells (Shi, 2017). The underlying mechanisms for selective expression of COX-2 in SMC by mechanical stretch are not yet clear. A study by Choudhury et al. (2015) has shown that mechano-transcription of COX-2 in the colon SMC was abrogated by de-polymerization of actin filament network with latrunculin B or swinholide. Mechano-transcription of COX-2 was also markedly attenuated by siRNA silencing of SMC specific α-actin (Acta2). These data indicate that SMC specific α-actin within an intact actin filament network is essential in the process whereby mechanical stress induces up-regulation of COX-2 in the colon SMC (Choudhury et al., 2015). Furthermore, Li et al. (2012a) reported that mechanical stretch potently activates mitogen-activated protein kinase (MAPK) p38 and protein kinase C (PKC), which are crucial intracellular signaling pathways involved in mechano-transcription of COX-2. However, treatments with Acta2 siRNA or latrunculin B or swinholide also restricted stretch-induced activation of p38 and PKC (Choudhury et al., 2015). Thus, an integrated smooth muscle actin network is essential for linking the mechanical signal to activation of MAPK and PKC and, thus, for mechano-transcription of COX-2.

Lin et al. (2012a) studied the effect of lumen distension on COX-2 expression in different parts of the GI tract by placing an obstruction band in the lower esophagus, pylorus, ileum, and colon. They depicted a significantly upregulated expression of COX-2 associated with obstruction at all the sites where distention was introduced (Lin et al., 2012a). They also found that COX-2 expression was significantly elevated by lumen distension of the colon with a balloon at a pressure of 40 mmHg for 40 min. Induction of COX-2 gene expression did not occur when the pressure was set 10 mmHg or less, or when the distention period was 20 min or shorter (Lin et al., 2015). Together, these studies demonstrate that distention-induced mechano-transcription of COX-2 in the GI tract is a force- and time-dependent, and smooth muscle specific phenomenon. Furthermore, mechano-transcription is a common process in response to luminal distention throughout the GI tract (Shi, 2017).

### Signal Transduction Mechanisms of Mechano-Transcription in the Gut

**FIGURE 3:**
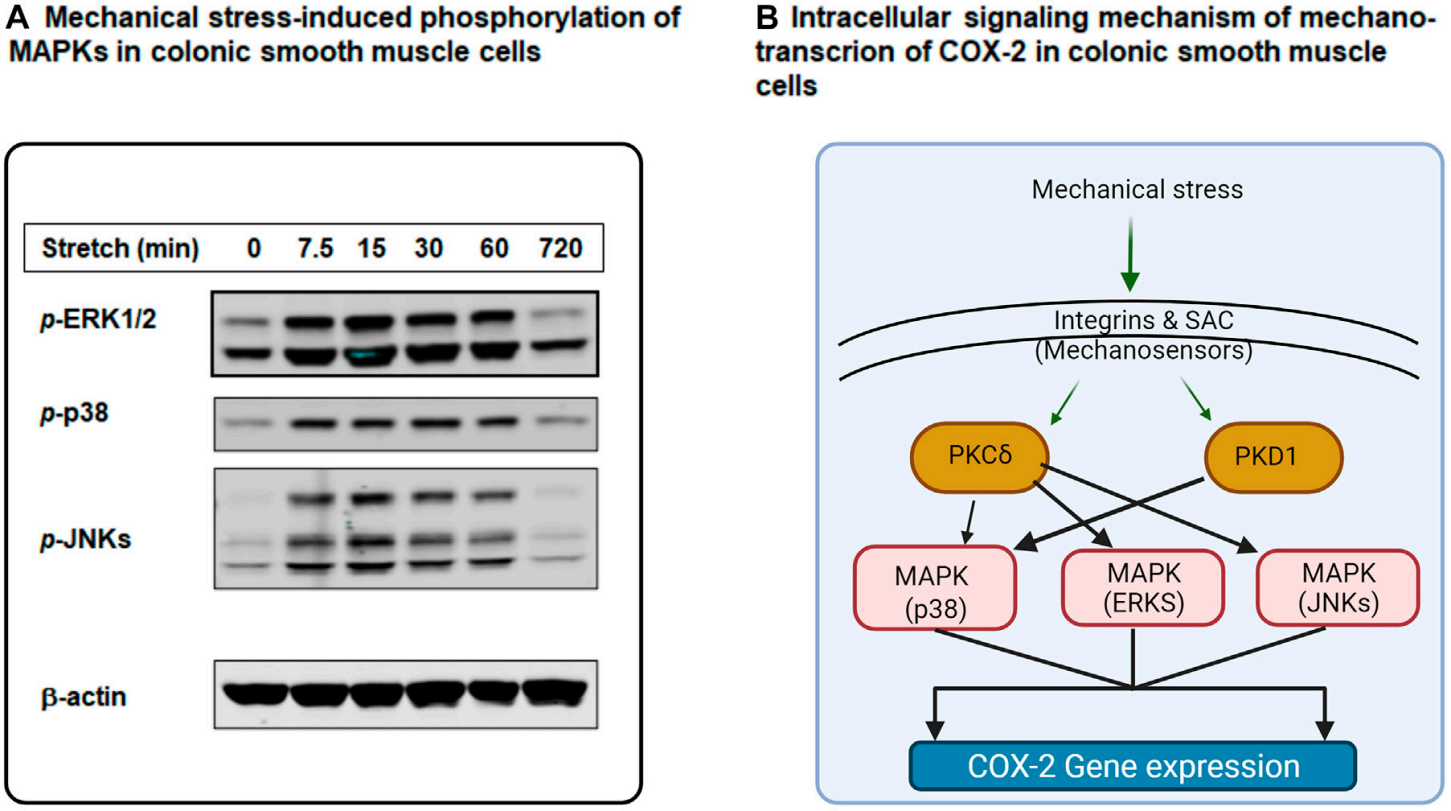
Intracellular signaling mechanisms of mechano-transcription of COX-2 in colonic smooth muscle cells (SMC). **(A)** Mechanical stretch (18%) induced robust phosphorylation of MAPKs ERK1/2, p38, and JNKs in cultured rat colonic SMC in a time-dependent manner. **(B)** Mechanical stimulation on gut SMC is sensed by integrins and SAC (stretch-activated ion channels) at the cell membrane level. These mechano-sensors transduce mechanical signals to intracellular signaling pathways involving PKC (i.e., PKCδ), PKD (PKD1), and MAPKs (p38, ERKs, and JNKs), leading to mechano-transcription of COX-2 in colonic SMC. More specifically, PKCδ is linked to MAPKs ERKs, p38, and JNKs, whereas PKD1 is coupled to MAPK p38. Some parts of the figure are adapted with permission from Figure 2 of Shi (2017).

Mechano-transcription of COX-2 in the gut has important pathophysiological significance in obstructive conditions (Shi et al., 2011; Lin et al., 2017). We then studied the signaling mechanisms whereby mechanical stress induces gene expression of COX-2 in colonic SMC (Li et al., 2012a; Li et al., 2012b). COX-2 gene expression was increased dramatically by static mechanical stretch (18% elongation) of cultured rat colonic SMC. Phosphorylation of MAPKs including extracellular signal-regulated kinases (ERKs, i.e., ERK 1 and ERK2), p38, and c-Jun N-terminal kinases (JNKs) (Li et al., 2012a) was also markedly induced by mechanical stress (Figure 3A). Treatment of the cells with MAPK inhibitors to ERK (PD98059), to p38 (SB203580) or to JNKs (SP600125) all significantly inhibited mechano-transcription of COX-2. These studies demonstrate the critical role of MAPK members in the regulation of mechano-transcription of COX-2 in colonic SMC (Li et al., 2012a; Shi, 2017).

Generally, mechanical stimuli are sensed at the cell membrane level and then get transduced into the cytoplasm and nucleus (Ruwhof and van der Laarse, 2000; Adam et al., 2004; Yamaguchi, 2004; Kanefsky et al., 2006). Among all possible mechano-sensors, integrins and stretch-activated ion channels (SACs) are two main groups which are identified in various types of cells (Hu and Sachs, 1997; Gillespie and Walker 2001; Katsumi et al., 2004; Kraichely and Farrugia, 2007). Our lab investigated whether ανβ3, a major type of integrin in gut smooth muscle cells, is a mechano-sensor involved in mechano-transcription of COX-2 in rat colon SMC (Shi, 2017). Li et al. (2012a) found that stretch-induced expression of COX-2 mRNA and protein was abrogated by ανβ3 inhibitor echistatin, and by neutralizing antibody against rat β3. Further, echistatin treatment has significantly inhibited stretch-induced activation of p38, but not ERKs and JNKs. The downstream pathway of integrin signaling was found to be also important in mechano-transcription of COX-2 in gut SMC, as knockdown of integrin-linked kinase and focal adhesion kinase with specific siRNAs significantly suppressed mechano-transcription of COX-2 in the cells (Li et al., 2012a). Li et al. (2012a) further examined if SACs play a role in mechano-transcription of COX-2 in the colon SMC. General SAC inhibitor gadolinium (Ducret et al., 2010) or specific SAC blocker GsMTx-4 (Suchyna et al., 2000) dramatically attenuated mechanical stretch-induced expression of COX-2 mRNA and protein. Treatments with gadolinium or GsMTx-4 also inhibited stretch-induced phosphorylation of all MAPKs, i.e., ERKs, p38, and JNKs (Li et al., 2012a).

The potential role of protein kinase C (PKC) and protein kinase D (PKD) in mechano-transcription of COX-2 was also investigated. Mechanical stretch leads to robust activation of PKCs and PKDs in colonic SMC (Li et al., 2012b). While blockade of PKC-β or PKC-ζ could not restrict stretch-induced expression of COX-2, rottlerin, a specific PKC-δ inhibitor, almost completely inhibited stretch-induced COX-2 expression. PKD inhibitor CID755673 or siRNA silencing of PKD also significantly inhibited stretch-induced COX-2 expression. Interestingly, Rottlerin treatment attenuated mechanical stretch-induced activation of all MAPKs (ERKs, p38, and JNKs), whereas CID755673 solely restricted activation of p38 (Li et al., 2012b).

Together, recent studies have demonstrated that mechanical stress on gut SMC is sensed by integrins and SAC at the cell membrane level. Then, the mechanical signal is transduced to the cytoplasm *via* intracellular signaling molecules PKCs, PKD, and MAPKs to induce gene expression of COX-2 in colonic SMCs (Li et al., 2012a; Li et al., 2012b). Further, studies suggest that PKC-δ is linked to MAPKs ERKs, p38, and JNKs, whereas PKD is connected to MAPK p38 (Li et al., 2012a; Li et al., 2012b; Shi, 2017) (Figure 3B).

## Pathogenic Role of Mechano-Transcription in Gastrointestinal Disorders

### Motility Dysfunction

Motility dysfunction is well documented in obstructive conditions. Motor activity is increased immediately in the distended segment in bowel obstruction, and gradually decreased within hours (Fraser et al., 1980; Summers, 1999; Bertoni et al., 2004; Won et al., 2006). The initial phase of hyper-motility in the distended bowel is considered to be a physiological adaptation to the obstruction of food passage (Summers, 1999). However, the chronic suppression of motor activity is the biggest concern in patients with obstructive disorders, especially in chronic partial obstruction (Ripamonti and Mercadante, 2004), malignant obstruction (Ripamonti et al., 2001; Roeland and von Gunten, 2009), and functional obstruction (Thompson, 2006; Nunez et al., 2009; De Giorgio et al., 2011). The consequences of impaired motor activity include distention, nausea, vomiting, and constipation (Russell and Welch, 1990; Summers, 1999; Shi et al., 2011).

As COX-2 and COX-2-derived prostaglandins profoundly affect motility function (Krause and DuBois, 2000; Fornai et al., 2005), we investigated whether mechanical stress-induced expression of COX-2 and production of prostaglandins play a role in the sustained suppression of motility in obstruction. Our studies have revealed that smooth muscle contractility of the isolated circular muscle strips was suppressed dramatically at 24 h after obstruction, which continued through the 7-days course of obstruction (Shi et al., 2011; Li et al., 2012a). Importantly, pretreatment of the muscle strips isolated from BO rats with COX-2 inhibitor NS-398 restored muscle contractility. Moreover, PGE_2_ levels were nearly 15-fold higher in the medium of BO strips than that of the sham strips, and NS-398 treatment significantly lowered the PGE_2_ levels (Shi et al., 2011). Studies also revealed that colon obstruction led to marked induction of COX-2 and decrease of muscle contractility in wild-type mice (Shi et al., 2011). However, smooth muscle contractility was largely unaffected by obstruction in the COX-2 deficient mice (Shi et al., 2011). These studies indicate that stretch-induced expression of COX-2 and associated release of prostaglandins play a prominent role in the suppression of smooth muscle contractility during BO.

Recent reports suggest that induction of several other pro-inflammatory molecules by mechanical stress may also contribute to motility abnormalities (Wehner et al., 2010; Lin et al., 2014b; Docsa et al., 2020). It was discovered that mechanical stress *in vitro* in cultured gut SMCs or *in vivo* in the model of obstruction significantly induced gene expression of IL-6, chemokine (C-C motif) ligand 2 (CCL-2), iNOS, and several other pro-inflammatory mediators in the muscle cells. These molecules are known to not only play critical roles in inflammation but also lead to motility dysfunction in the gut (Lin et al., 2014b). Docsa et al. (2020) found that mechanical stress-induced chemokine (C-X-C motif) ligand 1 (CXCL-1) in macrophages suppressed intestinal smooth muscle contractility and may account for motility dysfunction in ileus, a common functional obstruction in post-operative period. Moreover, conditioned media of stretched muscle strips induces NF-κB activation, further increasing production of pro-inflammatory mediators. Thus, mechanical stress-induced pro-inflammatory mediators may contribute significantly to motility dysfunction in obstructive conditions.

Gut motility is subject to control by neurotransmitters and gut hormones. Mechanical stress is well known to alter release of neurotransmitters and gut hormones (Brierley and Blackshaw, 2006; Linan-Rico et al., 2016). However, it is not well known whether mechanical stress is involved in the regulation of synthesis or transcription (i.e., mechano-transcription) of gut hormones and neurotransmitters. Serotonin (5-HT) as a hormone and neurotransmitter in the gut plays an important role in mediating peristalsis in physiological status and motility dysfunction in varies G.I. pathologies (Gershon and Tack, 2007; Linan-Rico et al., 2016). Lumen distention certainly increases release of 5-HT from enterochromaffin (EC) cells in the mucosa layer. The levels of 5-HT in the local gut tissues appear to be increased chronically in gut inflammation and increased 5-HT may contribute to inflammation and motility dysfunction in inflammatory conditions such as Crohn’s disease and ulcerative colitis (Gershon and Tack, 2007; Linan-Rico et al., 2016; Terry and Margolis, 2017). What accounts for the chronic increase of 5-HT in the gut is not well understood. However, a recent study found that cyclic mechanical stretch *in vitro* increased transcription and activation of tryptophan hydroxylase-1 and vesicular monoamine transporter-1 and enhanced the release of 5-HT in the cultured enterochromaffin cells (Chin et al., 2012). Tryptophan hydroxylase-1 and vesicular monoamine transporter-1 are well involved in the synthesis and transport of 5-HT in the enterochromaffin cells. Given that expression of tryptophan hydroxylase-1 and vesicular monoamine transporter-1 is mechanically responsive and mechanical stress is well present in gut inflammation, it is possible that mechano-transcription may be involved in the increased production of 5-HT in GI pathologies such as inflammation. Further studies are warranted to determine if mechano-transcription plays a role in the production of other gut hormones and neurotransmitters to contribute to motility dysfunction and inflammation.

### Abdominal Pain

Abdominal pain is a common complaint in obstructive conditions as well as functional bowel disorders and inflammatory bowel disease (Shi et al., 2018). Mechanical stimulation leads to instant activation of sensory nerves *via* mechanosensitive channels (Ness et al., 1990; Brierley and Blackshaw, 2006; Lin et al., 2015). This may contribute to the mechanisms involved in transmission of acute pain during early hours of obstructive conditions (Summers, Yanda, 1983; Shi et al., 2018). However, neuronal desensitization may occur upon repetitive or long-term mechanical stimulation (Slugg et al., 2000; Brierley et al., 2004). Nevertheless, chronic abdominal pain is present in obstruction (Ripamonti et al., 2001; Ripamonti and Mercadante, 2004; Roeland and von Gunten, 2009). Reports suggest that more than 90% of patients with advanced malignant obstruction have distention-associated abdominal pain (Baines et al., 1985; Ripamonti and Mercadante, 2004). Studies found that visceral sensitivity is markedly increased in chronic bowel obstruction (Huang et al., 2005; Lin et al., 2017). Visceral hypersensitivity is the main mechanism of chronic abdominal pain (Mayer and Gebhart, 1994; Bielefeldt, 2006; Azpiroz et al., 2007). We demonstrated that sensory neurons in the dorsal root ganglia (DRG) projecting to the obstructed colon in rats exhibited reduced resting membrane potential and rheobase along with increased number of action potentials (Lin et al., 2017). These studies demonstrate a highly sensitized state of the primary sensory afferents in obstruction (Lin et al., 2017). In addition, the withdrawal response to von Frey filament stimulation to the abdomen was found significantly increased in the rats with colon obstruction, suggesting referred visceral hyperalgesia (Lin et al., 2017).

Neurotrophins such as nerve growth factor (NGF) and brain-derived neurotrophic factor (BDNF) are well-known pain mediators (Bielefeldt, 2006; Pezet and McMahon, 2006). Recent studies found that expression of NGF and BDNF is highly responsive to mechanical stress (Lin et al., 2017; Fu et al., 2018). The expression of NGF mRNA and protein was significantly induced by mechanical stretch in colonic SMC *in vitro* and in bowel obstruction model (Lin et al., 2017). In the obstructed rats, the cell excitability of colon-projecting DRG neurons was augmented and the referred visceral sensitivity was increased. However, anti-NGF antibody administration largely restored the colon neuron excitability and referred visceral sensitivity in obstructed rats. Lin et al. (2017) observed that tetrodotoxin-resistant (TTX-r) Na^+^ currents and TTX-r Na_v_1.8 mRNA expression were significantly increased in colon-projecting DRG neurons in colon obstruction. However, the increased Na^+^ channel activity and Na_v_1.8 mRNA expression were attenuated by anti-NGF treatment. Therefore, mechanical stretch-induced NGF in colon SMC contributes significantly to visceral hypersensitivity in BO by sensitizing primary afferents and increasing TTX-r Na_v_ expression and function in the DRG neurons. Similarly, studies have revealed that expression of BDNF was also induced significantly in the colonic smooth muscle cells of the mechanically distended bowel segment in obstruction (Fu et al., 2018). The colon-projecting DRG neurons of the obstructed rats exhibited significantly reduced densities of voltage-gated K^+^ channel (K_v_) and transient A-type (*I*_*A*_) current. These changes contributed to neuronal hyper-excitability. Anti-BDNF antibody treatment blocked these changes in neurons isolated from obstructed rats. Administration of ANA-12, an inhibitor to BDNF receptor Trk B, also blocked the changes of neuronal excitability and K_v_ activity, and improved referred visceral sensitivity in obstructed rats (Fu et al., 2018). Together, these studies depict the critical contribution of mechano-transcription of NGF and BDNF in gut SMC to visceral hypersensitivity and abdominal pain in obstruction.

Visceral inputs are transduced to primary sensory neurons located in DRG, which further transmit the signals to the second order neurons in the spinal cord to initiate central processing of sensory information for perception (Lin et al., 2017; Shi et al., 2018). There is evidence that mechanical distension in the gut may affect gene expression and function of sensory pathway directly and indirectly. It is found that gene expression of opioid receptors in primary sensory afferents was down-regulated by mechanical stress during bowel obstruction, and down-regulation of the anti-pain system may contribute to visceral hypersensitivity in obstruction (Hegde et al., 2020). Moreover, persistent functional and transcriptional changes may occur in neurons in the DRG and dorsal horn secondary to pro-inflammatory and pain mediators induced by mechano-transcription in the peripheral tissues after lumen distension (Lin et al., 2018 abstract). The membrane excitability of colon-specific DRG neurons remains significantly enhanced 14 days after a 7-days partial colon obstruction. The mRNA expression and channel activity of transient receptor potential cation channel subfamily V member 1 (TRPV1) are increased not only during obstruction, but 14 days after obstruction is released. The long-term change of TRPV1 gene expression may be secondary to the effect of mechanical stress-induced NGF in the obstructed colon, as NGF increases TRPV1 expression in DRG neurons (Winston et al., 2001).

### Gut Inflammation

Gut inflammatory milieu involves multiple cellular and molecular processes that are mediated by cytokines, chemokines, and other inflammatory mediators (Papadakis and Targan, 2000; Stadnyk, 2002). Recent studies have shown that mechanical force is a pro-inflammatory stimulus in the gut (Wehner et al., 2010; Lin et al., 2014b; Shi, 2017). Wehner et al. (2010) reported that static mechanical stress significantly induced iNOS, COX-2, and IL-1β gene expression levels in cultured gut SMCs and peritoneal macrophages. Mechanical stimulation also magnified lipopolysaccharide-induced iNOS and IL-1 gene expression in intestinal smooth muscle cells, and similarly COX-2 and IL-6 mRNA expression in macrophages. Lin et al. (2014b) further investigated mechano-sensitive expression of pro-inflammatory mediators in the gut with a comprehensive approach involving *in vitro*, *in vivo*, and *ex vivo* models. In the primary culture of colon smooth muscle cells, they found that static stretch significantly increased mRNA expression of iNOS, COX-2, IL-6, and CCL-2 (Lin et al., 2014b). Mechanical stretch did not have an effect on gene expression of TNF-α, IL-1β, and IL-8. In the *in vivo* model of partial colon obstruction, the authors found that expression of iNOS, IL-6, and CCL-2 is significantly increased in a time-dependent way in the mechanically distended segment, compared to the non-distended segment of the rats with obstruction or sham control animals. The conditioned media from the stretched colon smooth muscle significantly induced translocation and phosphorylation of pro-inflammatory transcription factor NF-κB p65, leading to increased mRNA expression of more inflammatory mediators in naïve cells. However, treatment with IL-6 neutralizing antibody to the conditioned medium from the mechanically distended muscle showed significant reduction in the activation of NF-κB and gene expression of inflammatory mediators, indicating a critical role of mechanical stress-induced IL-6 in the secondary activation of pro-inflammatory transcription factors (Lin et al., 2014b).

Osteopontin (OPN) is a secreted glycoprotein with many demonstrated roles in the regulation of immune response on multiple levels (Uede, 2011; Rittling and Singh, 2015). Sato et al. (2005) found that active Crohn’s disease (CD) patients demonstrated significantly higher plasma OPN levels than normal or ulcerative colitis (UC) patients, and the elevated plasma OPN levels in CD patients were significantly correlated with disease activity. Further, OPN was found to facilitate production of IL-12 from lamina propria mononuclear cells and is tightly involved in the Th1 immune response in CD (Ashkar et al., 2000; Sato et al., 2005). However, the cellular source and mechanisms of increased plasma OPN in CD are not clear. We found that expression of OPN was dramatically upregulated in the mechanically distended colon in bowel obstruction (Lin and Shi, 2021). OPN level was also increased in the plasma in rats with bowel obstruction. In the rat model of stenotic Crohn’s colitis, OPN expression was found increased not only at the inflammation site, but at the distended pre-inflammation site (Lin and Shi, 2021). Plasma OPN level was significantly increased in stenotic Crohn’s colitis rats. However, prevention of inflammation-associated mechanical distention with liquid diet eliminated OPN expression in the pre-inflammation site and normalized plasma OPN level. These results suggest that OPN expression in Crohn’s colitis is largely mediated by mechanical stress, and the plasma OPN levels in colitis are closely related to the extent of bowel distention.

### Intestinal Fibrosis and Smooth Muscle Hyperplasia

Stricture formation due to tissue fibrosis and smooth muscle hyperplasia is the constant challenge in CD (Li and Kuemmerle, 2014; Latella and Rieder, 2017; Rieder et al., 2017). Stricture-associated stenosis leads to bowel obstruction and increases risks of perforation and fistula (Katsanos et al., 2010; Latella and Rieder, 2017). Conventional treatments like anti-inflammatory agents are not much effective to prevent stricture formation in CD. Although surgery provides temporary relief, the previously distended pre-stenotic region may become new sites for recurrent inflammation and fibrosis. In fact, post-surgery endoscopic or histological recurrences are almost 100%, given enough time (Rutgeerts et al., 1991; Olaison et al., 1992; Rieder et al., 2017). Release of mechanical stress by strictureplasty reduces fibrosis (Yamamoto et al., 2007; Latella and Rieder, 2017). These clinical findings indicate that mechanical stress, as an inflammation-independent factor, may play a critical role in fibrosis and stricture formation (Rieder et al., 2017; Tschumperlin et al., 2018).

**FIGURE 4:**
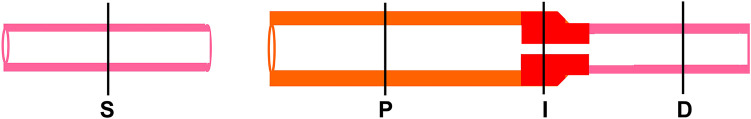
Diagram of sham and CD-like colons showing different sites. S, sham colon. Site I, the inflammation site, is present with inflammation and mechanical stress. Site P is the mechanically distended colon site proximal to inflammation. It shows no sign of inflammation. Site D, the non-distended site distal to inflammation, presents neither mechanical stress nor inflammation.

In fact, there is increasing evidence that mechanical stress induces gene expression of pro-fibrotic and proliferative mediators to contribute to intestinal fibrosis and hyperplasia in the gut (Johnson et al., 2013; Johnson et al., 2014; Lin et al., 2021a). In a rodent model of Crohn’s-like colitis, Lin et al. (2021a) observed that intracolonic instillation of TNBS resulted in induction of localized transmural inflammation in the distal colon (represented as site I), with a distended colon segment (represented as site P) proximal to the inflammation site, and a non-distended segment (represented as site D) distal to the site of inflammation (Figure 4). Based on macroscopic and histological studies, site I comprises inflammation and mechanical stress, site P has mechanical stress but no visible inflammation, and site D has neither inflammation nor mechanical stress. Significant fibrosis and smooth muscle hyperplasia developed 7–21 days after TNBS instillation at sites P and I. Molecular expression analysis revealed that expression of pro-fibrotic and proliferative genes, e.g., connective tissue growth factor (CTGF) and BDNF were markedly up-regulated in both sites P and I. The increased expression of CTGF and BDNF were mainly observed in the muscularis externa. Interestingly, expression of CTGF and BDNF was not significantly upregulated in the non-distended site D, suggesting that expression of CTGF and BDNF expression is stretch-sensitive in the CD-like colitis model. However, if rats were fed exclusively with clear liquid diet, mechanical distention in sites P and I was prevented in the TNBS-treated rats. Moreover, up-regulation of CTGF and BDNF was also prevented in sites P and I. Treatment with clear liquid diet markedly improved inflammation, fibrosis, and muscle hyperplasia in the colitis rats. In the model of partial colon obstruction, where mechanical distention, but no inflammation, was induced by an obstruction band wrapped in the distal colon, the authors found that collagen production, smooth muscle cell numbers and thickness, and expression of CTGF and BDNF were all increased only in the distended segment prior to obstruction, but not in the non-distended distal colon (Lin et al., 2021a). Taken together, these studies suggest that transmural inflammation in CD-like colitis is associated with mechanical stress in the inflammation site and the distended segment proximal to inflammation, which leads to increased expression of pro-fibrotic and proliferative mediators. Mechanical stress-induced up-regulation of pro-fibrotic and proliferative mediators may contribute significantly to the development of fibrosis and muscle hyperplasia in CD.

## Targeting Mechano-Transcription Process for Therapeutic Potentials in Obstructive Bowel Disorders

### Challenges in the Management of Obstructive Bowel Disorders

Obstructions in the small and large intestines accounts for approximately 15% of hospital admissions for acute abdomen in the U.S. The management strategy for bowel obstruction varies depending on etiology and site of obstruction, and whether patients have a partial, complete or complicated obstruction, and whether they have previous abdominal surgery (Frago et al., 2014; Catena et al., 2019). Recent studies found that only 20% of bowel obstruction cases need acute surgical care, and most of patients with bowel obstruction are treated with non-operative management (Catena et al., 2019). This is particularly true for malignant bowel obstruction (MBO), which is a common complication in patients with bowel or gynecological cancers. Conservative management for BO patients includes nil per os, nasogastric suction, stenting, fluid replacement therapy, and medication treatments for various symptoms related to obstruction (Catena et al., 2019). Nasogastric drainage is generally only a temporary measure. Among medical treatments, opioid analgesics are used for abdominal pain caused by obstruction. Although they may provide certain degree of pain relief, opioid analgesics cause significant gastrointestinal adverse effects such as constipation and fecal retention, which would worsen motility dysfunction in obstruction. Anti-secretory and anti-emetics may be used to control vomiting and abdominal distention (Ripamonti et al., 2001). However, the effect of medical treatments in bowel obstruction is limited as they do not specifically address mechanical stress - the root cause of functional abnormalities such as motility dysfunction and abdominal pain in obstructive disorders.

Currently, surgical treatment for bowel obstruction is to release blockage as in mechanical obstruction or remove constrictions as in Hirschsprung’s disease (HD). In either way, the distended oral segment is kept in the gut (Williams et al., 2005; Heuckeroth, 2018). However, many patients suffer bowel dysfunctions during obstruction and even after the obstruction is surgically resolved (Di Lorenzo et al., 2000; Fevang et al., 2004; Menezes et al., 2006; Catto-Smith et al., 2007; Jarvi et al., 2010). Nearly 70% of the children with a history of HD have long-term gastrointestinal symptoms such as incontinence, constipation, abdominal distention, and enterocolitis by 10 years after pull-through surgery (Di Lorenzo et al., 2000; Catto-Smith et al., 2007). Recent reports revealed that motility dysfunction and constipation continue through adulthood in 30% of patients after surgical release of HD-associated obstruction in childhood (Jarvi et al., 2010). In the upper gut, infantile pyloric stenosis causes gastric outlet obstruction. Gastric sensory and motility functions are affected for a long time even after pyloromyotomy (Sun et al., 2000; Saps and Bonilla, 2011). Dysregulated motility is accountable for symptoms such as bloating, nausea, vomiting, and constipation. However, the reasons for the long-term bowel dysfunction even after the resolution of obstruction is not understood. There are no specific treatments for the long-term dysfunction in such cases.

### Targeting Mechano-Transcription of COX-2 and Prostaglandin E Synthase for Motility Dysfunction in Bowel Obstruction and After Obstruction Is Corrected

Given that mechanical stretch induces marked expression of COX-2, and COX-2 derived prostaglandins suppress muscle contractions (Shi et al., 2011; Lin et al., 2012a), Lin et al. (2012b) studied the therapeutic and prophylactic effects of COX-2 inhibitors on smooth muscle function and gut motility in bowel obstruction in rats (Lin et al., 2012b). COX-2 inhibitor NS-398 or vehicle was administered daily pre- and post-induction of obstruction to investigate its prophylactic and therapeutic efficacies, respectively. Obstruction led to significant decrease of muscle contractility and a very slow colonic transit rate. However, prophylactic treatment with NS-398, starting before obstruction is induced, significantly improved colonic transit and muscle contractility, and attenuated fecal retention in the obstructed colon. Even with the treatment of NS-398 starting day 3 post obstruction, the muscle contractility and colonic transit still improved by day 7. The team further investigated whether inhibition of PGE_2_ is beneficial in improving motility function in obstruction (Lin et al., 2012b). Four PGE_2_ receptors, EP1 to EP4, were identified in the rat colonic smooth muscle cells. Although treatments with EP1 and EP3 antagonists decreased normal muscle contractility in tissues taken from sham controls, they did not improve muscle contractility in tissues taken from obstructed colon. On the other hand, the EP2 and EP4 antagonists did not significantly affect control tissue, but restored muscle contractility in obstruction (Lin et al., 2012b). These studies demonstrated that PGE_2_ and its receptors EP2 and EP4 are specifically implicated in motility dysfunction mediated by mechanical stress-induced COX-2 in obstruction. EP2 and EP4 antagonists, along with COX-2 inhibitors, may have therapeutic potential in medical treatment of motility dysfunction in bowel obstruction.

Clinical studies support the pre-clinical findings that selective inhibition of COX-2 has benefits in treating obstructive bowel disorders. Retrospective studies in large cohort of patients and clinical trials found that the use of COX-2 inhibitor celecoxib decreased the paralytic ileus rates and did not result in any significant morbidity (Wattchow et al., 2009; Raju et al., 2015). A multicenter, blinded, and randomized clinical trial study found that selective COX-2 inhibitor firocoxib, but not the nonselective COX inhibitor flunixin meglumine, is beneficial in small intestinal obstruction by strangulation in horses, as it reduced endotoxemia (Ziegler et al., 2019; Ziegler and Blikslager, 2020).

As BO-associated up-regulation of COX-2 and production of PGE_2_ depend on mechano-transcription process, Li *et al.* further determined whether inhibition of mechano-transcription signal transduction improves motility function (Li et al., 2012a; Li et al., 2012b). Because stretch-induced expression of COX-2 depends on MAPK p38 activation in colonic SMC, Li et al. (2012a) studied the *in vivo* effects of p38 inhibitor SB203580 on COX-2 induction and motility dysfunction in obstruction. It was found that SB203580 significantly inhibited induction of COX-2 and improved colon motility in obstruction. This was associated with improvement of colon distension. These studies show that inhibition of the intestinal mechano-transcription process has therapeutic potentials for motility dysfunction in obstructive bowel disorders.

Gut motility is altered not only during BO, but for a long time even after BO is resolved (Di Lorenzo et al., 2000; Fevang et al., 2004; Jarvi et al., 2010). Recent studies suggest that mechanical stress-induced COX-2 as well as COX-2–derived PGE_2_ in the distended gut SMC not just account for motility dysfunction in obstruction, but exert secondary effects on SMC even after obstruction is resolved (Lin et al., 2018). Lin et al. (2018) showed that COX-2–derived PGE_2_ during BO may further increase the expression of microsomal prostaglandin E synthase-1 (mPGES-1), an important enzyme involved in the synthesis of PGE_2_, in an autocrine mode. Studies *in vivo* showed that increased mPGES-1 contributes to the continuous production of PGE_2_ and long-term motility dysfunction. Thus, it is crucial to recognize that once the bowel is distended during obstruction, it may never be considered as “normal” because it is the site of mechano-transcription. Comparative studies were performed using COX-2 inhibitor NS-398 and mPGES-1 inhibitor Cay 10,526 during BO and post-BO (after obstruction is corrected). Results of the study suggest that targeting COX-2 and mPGES-1 during obstruction may be an effective therapeutic strategy to treat motility dysfunction during obstruction and a prophylactic strategy to prevent long-term motility dysfunction occurring after obstruction is resolved. However, for long-term motility dysfunction after the obstruction is corrected, inhibition of mPGES-1 rather than COX-2 appears to be a better therapeutic approach (Lin et al., 2018).

### Targeting Mechano-Regulation of Nociceptive Mediators and Anti-pain System for Abdominal Pain in Obstructive Bowel Disorders

Abdominal pain is the main reason for hospital visits in patients with bowel obstruction (Cappell and Batke, 2008; Gore et al., 2015). In the acute phase of obstruction (the first 12–24 h), abdominal pain may be colicky (cramping and intermittent) (Summers, 1999; Cartwright and Knudson, 2008; Gore et al., 2015). However, patients with chronic partial obstruction or pseudo-obstruction do experience persistent abdominal pain (Baines et al., 1985; Ripamonti et al., 2001; Ripamonti and Mercadante, 2004; De Giorgio et al., 2011). Currently available treatment for BO-associated pain relies on high doses of opioids (Ripamonti and Mercadante, 2004; Roeland and von Gunten, 2009). However, opioids are notorious in causing further motility dysfunction, constipation and paradoxically visceral hyperalgesia (Grunkemeier et al., 2007; Farmer et al., 2019; Lin et al., 2021b). It is imperative to identify visceral analgesics that are specific for distension-associated pain.

Recent studies have shown that mechanical stress induces marked expression of pain mediators NGF, BDNF, and COX-2 in the gut wall, which may play a crucial role in visceral hypersensitivity in experimental obstruction (Huang et al., 2005; Lin et al., 2015; Lin et al., 2017; Fu et al., 2018). Lin et al. (2017) found that inhibition of mechanical stress induced NGF by administering neutralizing antibody against NGF not only attenuated afferent neuron hyperexcitability, but also significantly improved pain behavior in rats with chronic partial colon obstruction. Fu et al. (2018) reported that BDNF is robustly induced by mechanical stress in distended bowel in obstruction, and blockade of BDNF action by administering Trk B inhibitor *in vivo* effectively inhibited BO-associated referred pain. These studies have also characterized downstream mechanisms of NGF and BDNF-mediated hyperalgesia and found that altered expression and activity of Na_v_1.8 and K_v_ in the afferent nerve may account for NGF and BDNF-mediated peripheral visceral hypersensitivity (Lin et al., 2017; Fu et al., 2018). Therefore, mechanical stress-induced production of NGF and BDNF in the gut tissues and the neurotrophins-mediated Na_v_ and K_v_ activity in primary afferent nerves represent plausible therapeutic targets for distention-associated abdominal pain.

Peripheral opioid receptors as a part of anti-pain system are thought to be critical in modulating visceral pain. It is found that while mechanical distention led to up-regulation of nociceptive mediators (e.g., NGF and BDNF) in gut SMC, the same distention caused down-regulation of opioid receptors *µ*, *δ*, and *κ* in the sensory afferents in the colon (Hegde et al., 2018). The down-regulation of opioid receptors may contribute to visceral hyperalgesia in obstruction. Interestingly, Hegde et al. (2020) found that the abundance of gut commensal *Lactobacillus reuteri* was drastically decreased in the obstructed bowel in rats. However, when *L. reuteri* rat strains were ingested via gavage (1 × 10^9^ colony-forming units/g daily starting 2 days before obstruction), the precision microbial therapy attenuated visceral hyperalgesia. Treatment with *L. reuteri* also diminished hyperexcitability of the DRG neurons projecting to the distended colon. Importantly, treatment with *L. reuteri* prevented the down-regulation of opioid receptors. In addition, treatment with peripheral opioid receptor antagonist naloxone methiodide eliminated the analgesic effect of *L. reuteri* in obstruction. Thus, *L. reuteri,* via inducing opioid receptors in the gut tissues, may have therapeutic potential in preventing lumen distension-associated visceral hypersalgesia and abdominal pain in obstructive conditions (Hegde et al., 2020).

## Targeting Mechano-Transcription Process for Therapeutic Potentials in Inflammatory Bowel Disease

### Challenges in the Management of Inflammatory Bowel Disease

The pathogenic mechanisms of Crohn’s disease (CD) and ulcerative colitis (UC) remain unknown, and cures are unavailable (Hendrickson et al., 2002; Sartor, 2006). Current therapies for IBD are designed to induce prolonged remission. Among the therapeutic options, 5-aminosalicylates are used mainly for mild active IBD (predominantly for UC), and for maintenance treatment in UC (Domènech, 2006). Corticosteroids are considered the mainstay for induction of remission in moderate to severe active inflammation in both UC and CD (Hendrickson et al., 2002; Domènech, 2006). Immunomodulators such as azathioprine and methotrexate are second-line treatment, mainly because of safety profile and economic costs. They are often used for maintenance therapy in both UC and CD (Domènech, 2006; Wehner et al., 2010; Kansal et al., 2013), or for patients with steroid refractoriness or dependency. The use of biologic agents, such as anti-TNFα chimeric antibody, is increasing. They are now often used as a first line therapy for moderate to severe CD and particularly useful in patients with steroid refractory disease (Ruemmele et al., 2009).

Unfortunately, most of the medical treatment options for IBD have substantial adverse effects. Corticosteroids are known for their frequent and sometimes severe side effects and their limitations include risks of infection, osteoporosis, growth retardation, poor mucosal healing, and early relapses on cessation of therapy (Sidoroff and Kolho, 2012; Cheifetz, 2013). This is especially problematic in pediatric patients, as this population of patients are prone to experience growth retardation and osteoporosis with steroid therapy (Sidoroff and Kolho, 2012). Immunomodulators increase the risk of opportunistic infections and hematologic disorders (Kandiel et al., 2005; Sidoroff and Kolho, 2012). Biologic agents targeting cytokines and adhesion molecules may lose efficacy over time, and are limited with increased vulnerability to infections, development of autoimmune disorders and even malignancy, and decreased immunogenicity of vaccinations (Kandiel et al., 2005). Thus, safe and effective therapies, including diet-based treatments, are much needed for IBD patients (Kansal et al., 2013; Ashton et al., 2019).

### Attenuation of Mechano-Transcription Process as a Novel Mechanism Underlying the Benefits of Exclusive Enteral Nutrition in Crohn’s Disease

Although many dietary therapies have been explored for the management of IBD (Domènech, 2006), exclusive enteral nutrition (EEN) is the sole established dietary treatment for IBD, specifically for CD (Kansal et al., 2013; Ashton et al., 2019). EEN involves oral or nasogastric tube feeding of a complete liquid diet with exclusion of normal foods for a defined period (usually 4–8 weeks). Over the last 2 decades, EEN has emerged as a highly effective treatment for the induction of remission in CD (Levine et al., 2018; Ashton et al., 2019). As a low-risk and steroid-sparing treatment, EEN is now the first-line therapy for pediatric CD patients. The reported remission rates with EEN treatment in this population of patients are up to 80% (Swaminath et al., 2017; Levine et al., 2018; Ashton et al., 2019). Consensus guidance from several organizations in Europe and North America (i.e., ECCO, ESPGHAN, ESPEN and NASPGHAN) directs clinicians to use EEN to induce remission in young CD patients wherever possible (Critch et al., 2012; Ruemmele et al., 2014; Forbes et al., 2017). It is suggested that corticosteroids, or early immunosuppressive therapy, should be reserved for the patients where EEN is not an option (Ruemmele et al., 2014; Ashton et al., 2019). There are reports that EEN is also effective in adult CD patients (Mowat et al., 2011; Yang et al., 2017). However, the evidence for the efficacy of EEN in adult patients is weaker than in pediatric population, possibly due to practicalities of its use (compliance, tolerability, etc.) (Ashton et al., 2019). Interestingly, there seems to be no established therapeutic role for EEN in the treatment of UC, an IBD that is without transmural inflammation and stricture formation as seen in CD (Ashton et al., 2019).

With EEN treatment, patients’ disease activity, mucosal healing, bowel symptoms, and nutrition status are all significantly improved (Ashton et al., 2019). Systemic inflammatory markers, such as C-reactive protein and erythrocyte sedimentation rate are normalized, and often corrected before any detectable change in nutrition status (Bannerjee et al., 2004; Grover et al., 2014). However, the exact mechanisms for the therapeutic benefits of EEN on inflammation and gut function in CD are still not well known, though several theories have been tested or postulated. Originally, elemental liquid diet (with amino acids, but no whole proteins) was first tested in CD as a means to provide nutritional support (Voitk et al., 1973). Subsequent open and randomized controlled trials confirmed the benefits of exclusive elemental liquid diet therapy in inducing remission in active CD (Axelsson and Jarnum, 1977; O’Moráin et al., 1984). Some suspected that a reduced chance of allergic reaction with the elemental liquid diet could be a possible reason for the efficacy. However, numerous follow-up studies found that nitrogen sources of enteral feeds are not relevant to their therapeutic efficacy. EEN treatments with either elemental or whole proteins-based polymeric liquid diets are equally effective in inducing remission in active CD (Giaffer et al., 1990; Verma et al., 2000; Zachos et al., 2007).

Many studies have attempted to examine if the EEN efficacy is attributed to improved gut microbiota. (Quince et al., 2015; Ashton et al., 2016; MacLellan et al., 2017). However, as all elemental and non-elemental diets are similarly effective in inducing remission of active CD, they lead to vastly different changes of microbiome in the gut (Ashton et al., 2016; Ashton et al., 2019). With EEN treatment, normal gut commensal bacteria (i.e. *Bacteriodes, Prevotella,* Enterobacteriaceae, etc.) have been reported to both increase and decrease in relative abundances (Leach et al., 2008; Kaakoush et al., 2015; Dunn et al., 2016). For instance, early studies found that *Faecalibacterium prausnitzii*, an anti-inflammatory commensal, was increased with EEN (Sokol et al., 2008); however more recent reports have shown a reduction of its abundance over the course of treatment with EEN (Jia et al., 2010). Systemic reviews of the most recent and well-documented studies suggest a paradoxical effect of EEN that it causes a reduction of bacterial diversity and richness and changes on the microbiome usually with a dysbiosis (Gatti et al., 2017; MacLellan et al., 2017). These changes are less likely to account for the benefits of EEN in CD, because reduced bacterial diversity and richness would predispose the gut to more inflammatory changes.

Inflammation in active CD is associated with increased intestinal permeability and damaged barrier function (Levine and Wine, 2013). Whether the increased permeability is the cause, or a consequence of CD is unknown. However, Sanderson et al. (1987) reported that EEN improved the abnormal permeability after 6 weeks of EEN treatment. *In vitro* studies on enterocytes found that EEN elemental formula components such as glutamine and vitamin D3 attenuated TNF-α-induced production of IL-8 but enhanced nitric oxide production in colonic epithelial cell line HT-29 (Alhagamhmad et al., 2017). However, this effect may not be due to the direct effects of elemental components, as polymeric diet and solid foods contain these components too, especially after they undergo mechanical and chemical digestions in the GI tract. Some studies observed that EEN treatment may directly reduce production of pro-inflammatory cytokines IL-6, IL-8, and TNF-α in the intestine (Heuschkel et al., 2000; Ashton et al., 2019). However, the mechanisms for the improved intestinal permeability and reduced cytokine production are not clear (Levine and Wine, 2013).

As discussed above, mechanical stress is an inevitable pathological change in gut inflammation, especially in CD where transmural inflammation and stenosis are present. Recent studies show that mechanical stress may contribute to the development of gut inflammation and dysfunction in IBD (Wehner et al., 2010; Lin et al., 2014b; Lin and Shi, 2021). We have evidence that the benefits of EEN in CD may be due to its action to reduce mechanical stress and attenuate mechano-transcription of pro-inflammatory mediators in the gut (Lin et al., 2021a; Lin and Shi, 2021). In the rat model of Crohn’s-like colitis induced by intra-colonic instillation of TNBS, EEN treatment with liquid diet (*Ensure*, Abbott) significantly improved body weight gain and reduced inflammation and disease activity in colitis rats. TNBS instillation induced a localized transmural inflammation with thickened wall and narrowed lumen in the distal colon and caused marked lumen distention with fecal retention in the segment proximal to the inflammation site (Lin et al., 2021a; Lin and Shi, 2021). However, EEN treatment eliminated lumen distention in the inflamed colon. Our study in the control rats with no TNBS instillation found that EEN treatment dramatically reduced fecal mass and pellet size, and increased water content of the fecal pellets. We found in TNBS colitis rats that mRNA expression of pro-inflammatory mediators such as IL-6, MCP-1, OPN and COX-2 was dramatically induced in the inflammation site (site I) and the distended segment proximal to inflammation (site P), but not in the non-distended segment distal to inflammation (site D). Strikingly, EEN treatment almost completely blocked the up-regulation of pro-inflammatory gene expression, suggesting that a mechanical stress-associated mechanism is involved in colitis-associated induction of pro-inflammatory genes (Lin et al., 2021a; Lin and Shi, 2021). Among these mechano-sensitive pro-inflammatory mediators, OPN is found to contribute to the development of IBD, particularly the Th1 immune response in Crohn’s disease (Ashkar et al., 2000; Sato et al., 2005). Other mechano-sensitive pro-inflammatory mediators such as IL-6, MCP-1, and COX-2 in site I and site P all are also involved in the inflammatory process in the gut (Please refer to Section 3). Therefore, mechanical stress in the inflammation site and the distended segment prior to inflammation may play a pathogenic role in inflammation and gut dysfunction independent of the intrinsic inflammatory process. The benefits of EEN in inducing remission of inflammation may largely depend on the effect of EEN to reduce mechanical stress and attenuate the mechano-transcription process in the inflammation site and the site prior to the site of inflammation.

### Targeting Mechano-Transcription Process for Fibrosis and Smooth Muscle Hyperplasia in Crohn’s Disease

Stricture formation is a hallmark of severe Crohn’s disease (CD), a B2 phenotype of CD in Montreal classification (Satsangi et al., 2006). The two main pathological changes involved in stricture formation are intestinal fibrosis and smooth muscle hyperplasia (Li and Kuemmerle, 2014; Latella and Rieder, 2017; Rieder et al., 2017). Currently, there is no effective medical treatment for the debilitating complication. Although anti-inflammatory treatments may be effective for active inflammation in CD, they do not appear to be effective in preventing or treating intestinal fibrosis and muscle hypertrophy.

Increasing evidence suggests that mechanical stress induces expression of pro-fibrotic mediators and extracellular matrix (ECM) proteins in gut smooth muscle cells. Gutierrez and Perr reported that static mechanical stretch significantly enhanced expression of transforming growth factor beta-1 (TGF-β1) mRNA and protein in intestinal smooth muscle cells. Expression of type 1 collagen mRNA and protein was also increased by mechanical stretch in these cells (Gutierrez and Perr, 1999). Recent *in vivo* studies in our lab have shown that mechanical stretch not only increases production of collagen expression and ECM deposition, but also induces robust expression of pro-fibrotic mediators such as CTGF in the colon (Lin et al., 2021a). Expression of CTGF is increased not only at the site of inflammation, but also in the distended site proximal to inflammation in TNBS-induced CD-like colitis. CTGF has long been recognized to have potent effect on cell proliferation and production of ECM proteins such as collagens. CTGF expression was found increased in CD and UC not only in the inflammation area, but also in areas of little inflammation but severe fibrosis (Dammeier et al., 1998; Di Mola et al., 2004). Our recent studies in rats have revealed that mechanical stress induced CTGF in gut SMC, which may contribute significantly to fibrosis. We inhibited CTGF by administering anti-CTGF antibody (FibroGen, San Francisco, CA). Comparing to IgG control, anti-CTGF treatment significantly attenuated fibrosis in the site of inflammation and site proximal to inflammation (Lin and Shi, unpublished observation). Our study suggests that mechanical stress-induced pro-fibrotic mediators such as CTGF may represent a potential therapeutic target in battling fibrosis in Crohn’s.

While fibrosis is a well-recognized change in stricture formation in CD, recent comprehensive histopathological analysis suggests that smooth muscle hyperplasia may be the most prominent histological change in fibrostenotic stricture in CD (Chen et al., 2017; Rieder et al., 2017). Mechanisms for smooth muscle hyperplasia in CD are not well understood. BDNF is known for its neurotrophic and nociceptive effects on neurons (Boesmans et al., 2008; Al-Qudah et al., 2014; Fu et al., 2018). However, most recent studies found that BDNF potently promotes SMC proliferation (Kwapiszewska et al., 2012; Freeman et al., 2017). Clinical studies found that BDNF expression is up-regulated in IBD tissues (Li et al., 2011; Steinkamp et al., 2012). We found that BDNF expression is highly responsive to mechanical stress in gut SMC (Fu et al., 2018), and BDNF is markedly induced in the inflammation site and pre-stenotic site in the CD rats (Lin et al., 2021a), as well as in the distended bowel in mechanical obstruction (Fu et al., 2018). Exogenous BDNF leads to robust proliferation of rat colon SMC. Anti-BDNF treatment or antagonist of BDNF receptor Trk B significantly reduces gut SMC proliferation (Lin and Shi, unpublished observation). These studies suggest that mechanical stress induced BDNF plays a key role in SMC hyperplasia in a preclinical model of CD and may be considered a potential therapeutic target for prevention or treatment of SMC hyperplasia and hypertrophy in fibro stenotic CD.

## Targeting Mechano-Transcription Process for Therapeutic Potentials in Functional Bowel Disorders

### Mechano-Transcription and Irritable Bowel Syndrome

Irritable bowel syndrome (IBS) is the most common and best described type of functional bowel disorders, affecting nearly 11% of the general population in the US (Lovell and Ford, 2012; Camilleri, 2014). Along with visceral pain, abdominal distention and bloating are major complaints among IBS patients (Chang et al., 2001a; Zar et al., 2002; Azpiroz and Malagelada, 2005; Shim et al., 2010). It is reported that 76% of IBS patients have abdominal bloating and 57% have abdominal distention (Chang et al., 2001b). The exact reasons of abdominal distention are not well understood. However, current evidence suggests that it may be ascribed to excessive gas accumulation and impaired gas transit in the gut (Koide et al., 2000; Serra et al., 2001; Hernando-Harder et al., 2010). Intraluminal retention of fluid and solid contents and altered gut microflora are also considered possible reasons for bloating and abdominal distention (Chang et al., 2001a; Agrawal et al., 2009; Sweetser et al., 2012). Some investigators found that ineffective evacuation, resulting in fecal retention in the colon and rectum, may well contribute to abdominal distention in IBS patients (Sweetser et al., 2012; Camilleri, 2014; Raahave and Jensen, 2021). Thus, abdominal distention in IBS is largely due to luminal retention of gas, liquid, or solid contents in the GI tract. The luminal retention of gas, liquid, and solid contents clearly represent mechanical stress to the gut wall [Lin et al., 2015).

As abdominal pain and distention are co-present in nearly 70% of IBS patients (Chang et al., 2001b), several groups have tried to determine whether distention contributes to abdominal pain. Earlier studies have focused on the immediate effect of repetitive distensions in the distal colon to noxious pressure levels (Ness et al., 1990; Gschossmann et al., 2001; Million et al., 2006). In general, a pressure is considered noxious when it is greater than 40 mmHg (Ness et al., 1990). In both humans and rats, repetitive colonic tonic distention (i.e., 60 mmHg for 10 min) resulted in visceral hypersensitivity (Ness et al., 1990; Gschossmann et al., 2001; Million et al., 2006). Moreover, luminal distention at noxious pressure (60 mmHg) induced changes in neuropeptide expression and ERK 1/2 activation in the dorsal horn (Lu et al., 2005; Million et al., 2006). These data suggest that colon distention at noxious pressure may induce acute visceral hypersensitivity *via* a central sensitization mechanism (Munakata et al., 1997; Lin et al., 2015; Shi et al., 2018).

Further studies found that colon distention may also contribute to chronic visceral hypersensitivity (Al-Chaer et al., 2000; Lin et al., 2015). Al-Chaer found that repetitive colon distention (60 mmHg) with a balloon in rats during neonatal stage led to visceral hypersensitivity detectable in adult stage and suggested that peripheral sensitization may be involved (Al-Chaer et al., 2000). Lin et al. (2015) tested colon distention in adult rats and found that tonic distention of the distal colon with a balloon at sub-noxious levels (20–40 mmHg) for 40 min led to significantly increased visceral sensitivity. The state of visceral hypersensitivity remained for at least 3 days. Electrophysiological studies showed that excitability of colon projecting sensory neurons in the dorsal root ganglia was significantly enhanced. Interestingly, they found that the sub-noxious mechanical distention induced expression of COX-2 and increased release of PGE_2_ in colonic muscularis externae, but not in the mucosa layer (Lin et al., 2015). Importantly, treatment with COX-2 inhibitor NS-398 abolished distention-induced production of PGE_2_, and significantly attenuated visceral hypersensitivity. These reports demonstrate that mechanical distention-induced production of inflammatory mediators, e.g., PGE_2_, contributes to visceral hypersensitivity in “functional” bowel disorders with lumen distention. PGE_2_ is found significantly increased in diarrhea-dominant IBS patients in the mucosa samples obtained by biopsy (Grabauskas et al., 2015), and is found to be the main cause of visceral hypersensitivity in IBS patients and animal models (Grabauskas et al., 2020). It is not known whether PGE_2_ is increased in IBS patients in the smooth muscle layer, which is the very site for mechanical stress-induced increase of COX-2 and PGE_2_. This is mainly because the smooth muscle layer is not accessible with a conventional biopsy. Nevertheless, COX-2 expression and PGE_2_ production were found significantly increased in smooth muscle layer of the colon obtained in surgery in patients with chronic constipation (Cong et al., 2007).

Several other mechanosensitive pain mediators like NGF and BDNF were found to be substantially increased in the bowel tissues of IBS patients compared to normal subjects (Yu et al., 2012; Dothel et al., 2015). These mediators are known to contribute to visceral hypersensitivity and abdominal pain in IBS (Dothel et al., 2015; Coelho et al., 2019). These studies did not specify the cause(s) for the increased expression of NGF and BDNF in the IBS tissues. However, mounting evidence suggests that expression of NGF and BDNF in the gut is highly inducible by mechanical stress, i.e., lumen distention (Lin et al., 2017; Fu et al., 2018). Thus, targeting mechano-transcription of inflammatory and pain mediators may have great therapeutic potentials for IBS (Shi et al., 2018).

### Mechano-Transcription and Other Functional Bowel Disorders

Among other functional bowel disorders, chronic constipation and fecal retention may present the most significant mechanical stress in the distal colon and rectum (Bharucha et al., 2006; Heredia et al., 2012; Raahave, 2015; Lin et al., 2021b). In chronic functional constipation, slowed colonic transit and decreased frequency of bowel movement directly lead to fecal accumulation or impaction in the distal bowel (Heredia et al., 2012; Lin et al., 2021b). As a separate entity of functional bowel disorders, fecal retention may be functional (voluntary withholding of stool) or due to obstructed defecation (Raahave, 2015; Raahave and Jensen, 2021). In either of the conditions, increased fecal accumulation in the distal bowel imposes apparent mechanical stretch to the bowel tissues, and thus may lead to mechano-transcription of select bioactive mediators (Lin et al., 2021b). A *Gastroenterology* study found that COX-2 expression and PGE_2_ production were significantly increased in the colonic muscularis externa tissues in patients with slow transit constipation (Cong et al., 2007). COX-2 expression was also found to be significantly up-regulated in the distended colon in slow transit constipation generated experimentally by outlet obstruction (Heredia et al., 2012), or by opioid-induced constipation (Lin et al., 2021b). Increased inhibitory prostaglandins such as PGE_2_ were found to be responsible for the diminished contractility in slow transit constipation in human (Liu et al., 2009) and in animal models (Heredia et al., 2012; Lin et al., 2021b). In fact, administration of COX-2 inhibitor NS-398 was found to be effective to improve bowel movement in constipation in chronic morphine treatment (Lin et al., 2021b). Fecal retention in the rodent model of opioid-induced bowel dysfunction also leads to significant increase of pain mediator NGF. Interventional studies suggest that the mechanical stress-induced pain mediators such as NGF may account for paradoxical hyperalgesia and narcotic bowel syndrome in chronic morphine treatment (Lin et al., 2021b).

Mechanical stress is also present in functional bowel disorders in the upper GI tract. In gastroparesis, for instance, slowed gastric emptying rate leads to increased retention of foods in the stomach as demonstrated in gastroparesis patients (Azpiroz et al., 2014; Steinsvik et al., 2021). When full thickness gastric tissues were available for studies, several groups reported that COX-2 expression was increased in the gastric tissues in animal models of gastroparesis induced by glucagon (Chen et al., 2017), lipopolysaccharide (West et al., 2006), or stress (Li et al., 2016). Partial obstruction of the gastric outlet leads to marked gastric retention and distention in the stomach, which is associated with dramatic induction of COX-2 (Lin et al., 2012a). Moreover, *in vivo* administration of COX-2 inhibitors improved gastric motor function in gastroparesis models (Lin et al., 2012a; Chen et al., 2017).

These studies suggest that mechanical stress-induced expression of COX-2 and production of prostaglandins may represent common potential targets for “functional” disorders with luminal distention such as constipation, fecal retention, and gastroparesis. Currently available medical treatments for these disorders rely on prokinetics or secretagogues to arbitrarily stimulate smooth muscle contractions or increase mucosal secretion (Bharucha et al., 2006; Sharma and Rao, 2017). Long term efficacy of these treatments has been compromised. However, inhibition of mechano-transcription process represents a promising therapeutic strategy, as it targets mechanical stress—perhaps the root cause of bowel dysfunctions in these disorders.

## Conclusion

**FIGURE 5:**
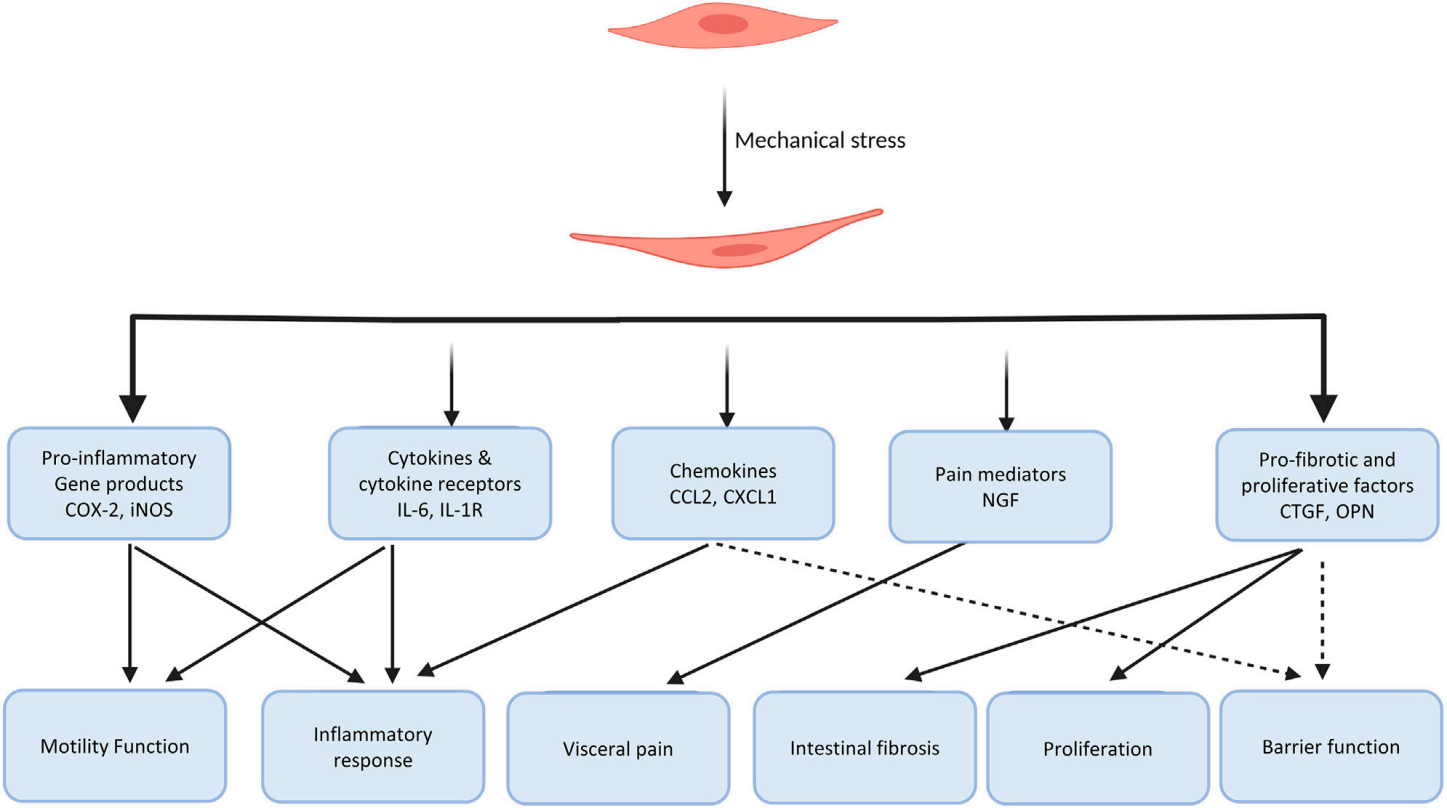
Diagram summarizing the gene products and pathophysiological roles of the mechano-transcription process in the gastrointestinal tract. Studies up to date have shown that mechanical stress-induced gene expression of pro-inflammatory molecules, chemokines, cytokines and their receptors, pain mediators, pro-fibrotic and growth factors play a critical role in pathogenesis of motility dysfunction, inflammatory response, visceral pain, intestinal fibrosis and cell proliferation in the gut. It is yet to determine if the mechano-transcription process is involved in secretion, permeability, and other functions and dysfunctions in the gut.

Mechanical stress, i.e., shear, stretch, and compression, is a common phenomenon encountered in the GI tract. Excessive mechanical stress is the root cause of obstructive conditions and is constitutively present in inflammatory conditions and some functional bowel disorders. Different tissues and cells in the GI tract respond differently to mechanical stress. In this review, we have focused on the recent discoveries of mechanical stress-altered gene expression, i.e., mechano-transcription, in the gut. Numerous *in vitro* and *in vivo* studies have demonstrated that mechanical stress profoundly alters gene expression in the GI tract especially smooth muscle cells. Mechano-transcription of pro-inflammatory molecules, pain mediators, fibrogenic and growth factors plays a critical role in motility dysfunction, abdominal pain, inflammation, fibrosis, and hyperplasia in various gastrointestinal disorders (Figure 5). We have shown pre-clinical and clinical evidence that mechanical stress-induced COX-2 and other pro-inflammatory mediators in gut smooth muscle cells account for motility dysfunction and inflammatory process in obstructive, inflammatory and some functional disorders. Mechanical stress-induced up-regulation of pain mediators such as nerve growth factor and brain-derived neurotrophic factor, and down-regulation of opioid receptors in the peripheral tissues may lead to visceral hypersensitivity. Emerging evidence suggests that mechanical stress in the gut also leads to up-regulation of certain genes encoding pro-fibrotic and proliferative mediators such as connective tissue growth factor and osteopontin, which may contribute to fibrostenotic Crohn’s disease. It is yet to determine whether the mechano-transcription process is involved in secretion, permeability, and other pathophysiological changes in the GI tract. Taken together, the studies over the last decade suggest that the mechano-transcription process may represent novel therapeutic targets for the management of obstructive, inflammatory, and functional bowel disorders. The strategy to target mechano-transcription process has a unique advantage, as it addresses one of the root causes (mechanical stress) of these disorders. Further studies of these targets are warranted before therapeutics can be developed for clinical use.

## References

[B1] Adam R. M. Eaton S. H. Estrada C. Nimgaonkar A. Shih S. C. Smith L. E. (2004). Mechanical Stretch Is a Highly Selective Regulator of Gene Expression in Human Bladder Smooth Muscle Cells. Physiol. Genomics 20, 36–44. 10.1152/physiolgenomics.00181.2004 15467014

[B2] Agrawal A. Houghton L. A. Reilly B. Morris J. Whorwell P. J. (2009). Bloating and Distension in Irritable Bowel Syndrome: the Role of Gastrointestinal Transit. Am. J. Gastroenterol. 104, 1998–2004. 10.1038/ajg.2009.251 19491831

[B3] Al-Chaer E. D. Kawasaki M. Pasricha P. J. (2000). A New Model of Chronic Visceral Hypersensitivity in Adult Rats Induced by colon Irritation during Postnatal Development. Gastroenterology 119, 1276–1285. 10.1053/gast.2000.19576 11054385

[B4] Al-Qudah M. Anderson C. D. Mahavadi S. Bradley Z. L. Akbarali H. I. Murthy K. S. (2014). Brain-derived Neurotrophic Factor Enhances Cholinergic Contraction of Longitudinal Muscle of Rabbit Intestine via Activation of Phospholipase C. Am. J. Physiol. Gastrointest. Liver Physiol. 306, G328–G337. 10.1152/ajpgi.00203.2013 24356881 PMC3920121

[B5] Alhagamhmad M. H. Day A. S. Lemberg D. A. Leach S. T. (2017). Exploring and Enhancing the Anti-inflammatory Properties of Polymeric Formula. JPEN J. Parenter. Enteral Nutr. 41, 436–445. 10.1177/0148607115625627 26826259

[B6] Ashkar S. Weber G. F. Panoutsakopoulou V. Sanchirico M. E. Jansson M. Zawaideh S. (2000). Eta-1 (Osteopontin): an Early Component of Type-1 (Cell-mediated) Immunity. Science 287, 860–864. 10.1126/science.287.5454.860 10657301

[B7] Ashton J. J. Beattie R. M. Ennis S. Cleary D. W. (2016). Analysis and Interpretation of the Human Microbiome. Inflamm. Bowel Dis. 22, 1713–1722. 10.1097/MIB.0000000000000809 27243592

[B8] Ashton J. J. Gavin J. Beattie R. M. (2019). Exclusive Enteral Nutrition in Crohn's Disease: Evidence and Practicalities. Clin. Nutr. 38, 80–89. 10.1016/j.clnu.2018.01.020 29398336

[B9] Axelsson C. Jarnum S. (1977). Assessment of the Therapeutic Value of an Elemental Diet in Chronic Inflammatory Bowel Disease. Scand. J. Gastroenterol. 12, 89–95. 10.1080/00365521.1977.12031117 834975

[B10] Azpiroz F. Bouin M. Camilleri M. Mayer E. A. Poitras P. Serra J. (2007). Mechanisms of Hypersensitivity in IBS and Functional Disorders. Neurogastroenterol Motil. 19, 62–88. 10.1111/j.1365-2982.2006.00875.x 17280586

[B11] Azpiroz F. Feinle-Bisset C. Grundy D. Tack J. (2014). Gastric Sensitivity and Reflexes: Basic Mechanisms Underlying Clinical Problems. J. Gastroenterol. 49, 206–218. 10.1007/s00535-013-0917-8 24306100

[B12] Azpiroz F. Malagelada J. R. (2005). Abdominal Bloating. Gastroenterology 129, 1060–1078. 10.1053/j.gastro.2005.06.062 16143143

[B13] Baines M. Oliver D. J. Carter R. L. (1985). Medical Management of Intestinal Obstruction in Patients with Advanced Malignant Disease. A Clinical and Pathological Study. Lancet 2, 990–993. 10.1016/s0140-6736(85)90534-3 2414614

[B14] Bannerjee K. Camacho-Hübner C. Babinska K. Dryhurst K. M. Edwards R. Savage M. O. (2004). Anti-inflammatory and Growth-Stimulating Effects Precede Nutritional Restitution during Enteral Feeding in Crohn Disease. J. Pediatr. Gastroenterol. Nutr. 38, 270–275. 10.1097/00005176-200403000-00007 15076624

[B15] Bertoni S. Gabella G. Ghizzardi P. Ballabeni V. Impicciatore M. Lagrasta C. (2004). Motor Responses of Rat Hypertrophic Intestine Following Chronic Obstruction. Neurogastroenterol Motil. 16, 365–374. 10.1111/j.1365-2982.2004.00510.x 15198659

[B16] Beyder A. (2018). In Pursuit of the Epithelial Mechanosensitivity Mechanisms. Front. Endocrinol. (Lausanne) 9, 804. 10.3389/fendo.2018.00804 30697191 PMC6340920

[B17] Bharucha A. E. Wald A. Enck P. Rao S. (2006). Functional Anorectal Disorders. Gastroenterology 130, 1510–1518. 10.1053/j.gastro.2005.11.064 16678564

[B18] Bielefeldt K. (2006). “Neurochemical and Molecular Basis of Peripheral Sensitization,” in Chronic Abdominal and Visceral Pain. Editors Pasricha P. J. Willis W. D. Gebhart G. F. (Boca Raton, FL: Informa Healthcare), 67–83.

[B19] Boesmans W. Gomes P. Janssens J. Tack J. Vanden Berghe P. (2008). Brain-derived Neurotrophic Factor Amplifies Neurotransmitter Responses and Promotes Synaptic Communication in the Enteric Nervous System. Gut 57, 314–322. 10.1136/gut.2007.131839 17965066

[B20] Brierley S. M. Jones R. C. 3rd Gebhart G. F. Blackshaw L. A. (2004). Splanchnic and Pelvic Mechanosensory Afferents Signal Different Qualities of Colonic Stimuli in Mice. Gastroenterology 127, 166–178. 10.1053/j.gastro.2004.04.008 15236183

[B21] Brierley S. M. Blackshaw L. A. (2006). “The Neurobiology of Visceral Nociceptors,” in Chronic Abdominal and Visceral Pain. Editors Pasricha P. J. Willis W. D. Gebhart G. F. (Boca Raton, FL: Informa Healthcare), 45–66.

[B22] Camilleri M. (2014). Physiological Underpinnings of Irritable Bowel Syndrome: Neurohormonal Mechanisms. J. Physiol. 592, 2967–2980. 10.1113/jphysiol.2014.270892 24665101 PMC4214653

[B23] Cappell M. S. Batke M. (2008). Mechanical Obstruction of the Small Bowel and colon. Med. Clin. North. Am. 92, 575–viii. 10.1016/j.mcna.2008.01.003 18387377

[B24] Cartwright S. L. Knudson M. P. (2008). Evaluation of Acute Abdominal Pain in Adults. Am. Fam. Physician 77, 971–978. 18441863

[B25] Catena F. De Simone B. Coccolini F. Di Saverio S. Sartelli M. Ansaloni L. (2019). Bowel Obstruction: a Narrative Review for All Physicians. World J. Emerg. Surg. 14, 20. 10.1186/s13017-019-0240-7 31168315 PMC6489175

[B26] Catto-Smith A. G. Trajanovska M. Taylor R. G. (2007). Long-term Continence after Surgery for Hirschsprung's Disease. J. Gastroenterol. Hepatol. 22, 2273–2282. 10.1111/j.1440-1746.2006.04750.x 18031392

[B27] Chang I. Y. Glasgow N. J. Takayama I. horiguchi K. Sanders K. M. Ward S. M. (2001a). Loss of Interstitial Cells of Cajal and Development of Electrical Dysfunction in Murine Small Bowel Obstruction. J. Physiol. 536, 555–568. 10.1111/j.1469-7793.2001.0555c.xd 11600689 PMC2278884

[B28] Chang L. Lee O. Y. Naliboff B. Schmulson M. Mayer E. A. (2001b). Sensation of Bloating and Visible Abdominal Distension in Patients with Irritable Bowel Syndrome. Am. J. Gastroenterol. 96, 3341–3347. 10.1111/j.1572-0241.2001.05336.x 11774947

[B29] Cheifetz A. S. (2013). Management of Active Crohn Disease. JAMA 309, 2150–2158. 10.1001/jama.2013.4466 23695484 PMC5877483

[B30] Chen W. Lu C. Hirota C. Iacucci M. Ghosh S. Gui X. (2017). Smooth Muscle Hyperplasia/hypertrophy Is the Most Prominent Histological Change in Crohn's Fibrostenosing Bowel Strictures: a Semiquantitative Analysis by Using a Novel Histological Grading Scheme. J. Crohns Colitis 11, 92–104. 10.1093/ecco-jcc/jjw126 27364949

[B31] Chin A. Svejda B. Gustafsson B. I. Granlund A. B. Sandvik A. K. Timberlake A. (2012). The Role of Mechanical Forces and Adenosine in the Regulation of Intestinal Enterochromaffin Cell Serotonin Secretion. Am. J. Physiol. Gastrointest. Liver Physiol. 302, G397–G405. 10.1152/ajpgi.00087.2011 22038827 PMC3287403

[B32] Choudhury B. Li F. Shi X. Z. (2015). Smooth Muscle Specific Alpha-Actin Plays a Critical Role in the Regulation of Mechanical Stress-Induced Gene Expression in the colon (Abstract). Gastroenterol 148, S536. 10.1016/s0016-5085(15)31798-4

[B33] Coelho A. Oliveira R. Antunes-Lopes T. Cruz C. D. (2019). Partners in Crime: NGF and BDNF in Visceral Dysfunction. Curr. Neuropharmacol 17, 1021–1038. 10.2174/1570159X17666190617095844 31204623 PMC7052822

[B34] Cong P. Pricolo V. Biancani P. Behar J. (2007). Abnormalities of Prostaglandins and Cyclooxygenase Enzymes in Female Patients with Slow-Transit Constipation. Gastroenterology 133, 445–453. 10.1053/j.gastro.2007.05.021 17681165

[B35] Cox C. S. Jr Radhakrishnan R. Villarrubia L. Xue H. Uray K. Gill B. S. (2008). Hypertonic saline Modulation of Intestinal Tissue Stress and Fluid Balance. Shock 29, 598–602. 10.1097/SHK.0b013e318157eba7 18414233

[B36] Critch J. Day A. S. Otley A. King-Moore C. Teitelbaum J. E. Shashidhar H. NASPGHAN IBD Committee (2012). Use of Enteral Nutrition for the Control of Intestinal Inflammation in Pediatric Crohn Disease. J. Pediatr. Gastroenterol. Nutr. 54, 298–305. 10.1097/MPG.0b013e318235b397 22002478

[B37] Dammeier J. Brauchle M. Falk W. Grotendorst G. R. Werner S. (1998). Connective Tissue Growth Factor: a Novel Regulator of Mucosal Repair and Fibrosis in Inflammatory Bowel Disease? Int. J. Biochem. Cel Biol 30, 909–922. 10.1016/s1357-2725(98)00046-6 9744082

[B38] De Giorgio R. Cogliandro R. F. Barbara G. Corinaldesi R. Stanghellini V. (2011). Chronic Intestinal Pseudo-obstruction: Clinical Features, Diagnosis, and Therapy. Gastroenterol. Clin. North. Am. 40, 787–807. 10.1016/j.gtc.2011.09.005 22100118

[B39] Di Lorenzo C. Solzi G. F. Flores A. F. Schwankovsky L. Hyman P. E. (2000). Colonic Motility after Surgery for Hirschsprung's Disease. Am. J. Gastroenterol. 95, 1759–1764. 10.1111/j.1572-0241.2000.02183.x 10925981

[B40] Di Mola F. F. Di Sebastiano P. Gardini A. Innocenti P. Zimmermann A. Büchler M. W. (2004). Differential Expression of Connective Tissue Growth Factor in Inflammatory Bowel Disease. Digestion 69, 245–253. 10.1159/000079845 15256831

[B41] Docsa T. Bhattarai D. Sipos A. Wade C. E. Cox C. S. Jr Uray K. (2020). CXCL1 Is Upregulated during the Development of Ileus Resulting in Decreased Intestinal Contractile Activity. Neurogastroenterol Motil. 32, e13757. 10.1111/nmo.13757 31722447

[B42] Domènech E. (2006). Inflammatory Bowel Disease: Current Therapeutic Options. Digestion 73 (Suppl. 1), 67–76. 10.1159/000089781 16498254

[B43] Dothel G. Barbaro M. R. Boudin H. Vasina V. Cremon C. Gargano L. (2015). Nerve Fiber Outgrowth Is Increased in the Intestinal Mucosa of Patients with Irritable Bowel Syndrome. Gastroenterology 148, 1002–e4. 10.1053/j.gastro.2015.01.042 25655556

[B44] Ducret T. El Arrouchi J. Courtois A. Quignard J. F. Marthan R. Savineau J. P. (2010). Stretch-activated Channels in Pulmonary Arterial Smooth Muscle Cells from Normoxic and Chronically Hypoxic Rats. Cell Calcium 48, 251–259. 10.1016/j.ceca.2010.09.011 21035852

[B45] Dunn K. A. Moore-Connors J. MacIntyre B. Stadnyk A. W. Thomas N. A. Noble A. (2016). Early Changes in Microbial Community Structure Are Associated with Sustained Remission after Nutritional Treatment of Pediatric Crohn's Disease. Inflamm. Bowel Dis. 22, 2853–2862. 10.1097/MIB.0000000000000956 27805918

[B46] Farmer A. D. Drewes A. M. Chiarioni G. De Giorgio R. O'Brien T. Morlion B. (2019). Pathophysiology and Management of Opioid-Induced Constipation: European Expert Consensus Statement. United Eur. Gastroenterol J 7, 7–20. 10.1177/2050640618818305 PMC6374852 30788113

[B47] Fevang B. T. Fevang J. Lie S. A. Søreide O. Svanes K. Viste A. (2004). Long-term Prognosis after Operation for Adhesive Small Bowel Obstruction. Ann. Surg. 240, 193–201. 10.1097/01.sla.0000132988.50122.de 15273540 PMC1356393

[B48] Forbes A. Escher J. Hébuterne X. Kłęk S. Krznaric Z. Schneider S. (2017). ESPEN Guideline: Clinical Nutrition in Inflammatory Bowel Disease. Clin. Nutr. 36, 321–347. 10.1016/j.clnu.2016.12.027 28131521

[B49] Fornai M. Blandizzi C. Colucci R. Antonioli L. Bernardini N. Segnani C. (2005). Role of Cyclooxygenases 1 and 2 in the Modulation of Neuromuscular Functions in the Distal colon of Humans and Mice. Gut 54, 608–616. 10.1136/gut.2004.053322 15831902 PMC1774510

[B50] Frago R. Ramirez E. Millan M. Kreisler E. del Valle E. Biondo S. (2014). Current Management of Acute Malignant Large Bowel Obstruction: a Systematic Review. Am. J. Surg. 207, 127–138. 10.1016/j.amjsurg.2013.07.027 24124659

[B51] Fraser I. D. Condon R. E. Schulte W. J. DeCosse J. J. Cowles V. E. (1980). Intestinal Motility Changes in Experimental Large Bowel Obstruction. Surgery 87, 677–682. 6769172

[B52] Freeman M. R. Sathish V. Manlove L. Wang S. Britt R. D. Jr Thompson M. A. (2017). Brain-derived Neurotrophic Factor and Airway Fibrosis in Asthma. Am. J. Physiol. Lung Cel Mol Physiol 313, L360–L370. 10.1152/ajplung.00580.2016 PMC5582935 28522569

[B53] Fu Y. Lin Y. M. Winston J. H. Radhakrishnan R. Huang L. M. Shi X. Z. (2018). Role of Brain-Derived Neurotrophic Factor in the Pathogenesis of Distention-Associated Abdominal Pain in Bowel Obstruction. Neurogastroenterol Motil. 30, e13373. 10.1111/nmo.13373 29781158 PMC6160336

[B54] Gabella G. (1975). Hypertrophy of Intestinal Smooth Muscle. Cell Tissue Res 163, 199–214. 10.1007/bf00221727 1182787

[B55] Gabella G. (1990). Hypertrophy of Visceral Smooth Muscle. Anat. Embryol. (Berl) 182, 409–424. 10.1007/BF00178906 2291488

[B56] Gatti S. Galeazzi T. Franceschini E. Annibali R. Albano V. Verma A. K. (2017). Effects of the Exclusive Enteral Nutrition on the Microbiota Profile of Patients with Crohn's Disease: a Systematic Review. Nutrients 9 (8), E832. 10.3390/nu9080832 28777338 PMC5579625

[B57] Gayer C. P. Basson M. D. (2009). The Effects of Mechanical Forces on Intestinal Physiology and Pathology. Cell Signal 21, 1237–1244. 10.1016/j.cellsig.2009.02.011 19249356 PMC2715958

[B58] Gershon M. D. Tack J. (2007). The Serotonin Signaling System: from Basic Understanding to Drug Development for Functional GI Disorders. Gastroenterology 132, 397–414. 10.1053/j.gastro.2006.11.002 17241888

[B59] Giaffer M. H. North G. Holdsworth C. D. (1990). Controlled Trial of Polymeric versus Elemental Diet in Treatment of Active Crohn's Disease. Lancet 335 (8693), 816–819. 10.1016/0140-6736(90)90936-y 1969560

[B60] Gillespie P. G. Walker R. G. (2001). Molecular Basis of Mechanosensory Transduction. Nature 413, 194–202. 10.1038/35093011 11557988

[B61] Gore R. M. Silvers R. I. Thakrar K. H. Wenzke D. R. Mehta U. K. Newmark G. M. (2015). Bowel Obstruction. Radiol. Clin. North. Am. 53, 1225–1240. 10.1016/j.rcl.2015.06.008 26526435

[B62] Grabauskas G. Wu X. Gao J. Li J. Y. Turgeon D. K. Owyang C. (2020). Prostaglandin E2, Produced by Mast Cells in Colon Tissues from Patients with Irritable Bowel Syndrome, Contributes to Visceral Hypersensitivity in Mice. Gastroenterology 158, 2195–e6. 10.1053/j.gastro.2020.02.022 32084424 PMC7282934

[B63] Grabauskas G. Wu X. Turgeon D. K. Li J. Y. Owyang C. (2015). Marked Elevation in Mucosal Proinflammatory PGE2 Is Responsible for Pain in Diarrhea-Predominant IBS (IBS-D) Patients. Gastroenterol 148 (Suppl. 1), S–775. 10.1016/s0016-5085(15)32642-1

[B64] Grover Z. Muir R. Lewindon P. (2014). Exclusive Enteral Nutrition Induces Early Clinical, Mucosal and Transmural Remission in Paediatric Crohn's Disease. J. Gastroenterol. 49, 638–645. 10.1007/s00535-013-0815-0 23636735

[B65] Grunkemeier D. M. Cassara J. E. Dalton C. B. Drossman D. A. (2007). The Narcotic Bowel Syndrome: Clinical Features, Pathophysiology, and Management. Clin. Gastroenterol. Hepatol. 5, 1126–1139. 10.1016/j.cgh.2007.06.013 17916540 PMC2074872

[B66] Gschossmann J. M. Coutinho S. V. Miller J. C. Huebel K. Naliboff B. Wong H. C. (2001). Involvement of Spinal Calcitonin Gene-Related Peptide in the Development of Acute Visceral Hyperalgesia in the Rat. Neurogastroenterol Motil. 13, 229–236. 10.1046/j.1365-2982.2001.00262.x 11437985

[B67] Gutierrez J. A. Perr H. A. (1999). Mechanical Stretch Modulates TGF-Beta1 and alpha1(I) Collagen Expression in Fetal Human Intestinal Smooth Muscle Cells. Am. J. Physiol. 277, G1074–G1080. 10.1152/ajpgi.1999.277.5.G1074 10564114

[B68] Hegde S. Lin Y. M. Fu Y. Savidge T. Shi X. Z. (2020). Precision Lactobacillus Reuteri Therapy Attenuates Luminal Distension-Associated Visceral Hypersensitivity by Inducing Peripheral Opioid Receptors in the colon. Pain 161, 2737–2749. 10.1097/j.pain.0000000000001967 32569084 PMC7669621

[B69] Hegde S. Lin Y. M. Golovko G. Khanipov K. Cong Y. Savidge T. (2018). Microbiota Dysbiosis and its Pathophysiological Significance in Bowel Obstruction. Sci. Rep. 8, 13044. 10.1038/s41598-018-31033-0 30177854 PMC6120911

[B70] Hendrickson B. A. Gokhale R. Cho J. H. (2002). Clinical Aspects and Pathophysiology of Inflammatory Bowel Disease. Clin. Microbiol. Rev. 15, 79–94. 10.1128/cmr.15.1.79-94.2002 11781268 PMC118061

[B71] Heredia D. J. Grainger N. McCann C. J. Smith T. K. (2012). Insights from a Novel Model of Slow-Transit Constipation Generated by Partial Outlet Obstruction in the Murine Large Intestine. Am. J. Physiol. Gastrointest. Liver Physiol. 303, G1004–G1016. 10.1152/ajpgi.00238.2012 22961801 PMC3517665

[B72] Hernando-Harder A. C. Serra J. Azpiroz F. Milà M. Aguadé S. Malagelada C. (2010). Colonic Responses to Gas Loads in Subgroups of Patients with Abdominal Bloating. Am. J. Gastroenterol. 105, 876–882. 10.1038/ajg.2010.75 20179685

[B73] Heuckeroth R. O. (2018). Hirschsprung Disease - Integrating Basic Science and Clinical Medicine to Improve Outcomes. Nat. Rev. Gastroenterol. Hepatol. 15, 152–167. 10.1038/nrgastro.2017.149 29300049

[B74] Heuschkel R. B. MacDonald T. T. Monteleone G. Bajaj-Elliott M. Smith J. A. Pender S. L. (2000). Imbalance of Stromelysin-1 and TIMP-1 in the Mucosal Lesions of Children with Inflammatory Bowel Disease. Gut 47, 57–62. 10.1136/gut.47.1.57 10861265 PMC1727949

[B75] Hu H. Sachs F. (1997). Stretch-activated Ion Channels in the Heart. J. Mol. Cel Cardiol 29, 1511–1523. 10.1006/jmcc.1997.0392 9220338

[B76] Huang T. Y. Hanani M. (2005). Morphological and Electrophysiological Changes in Mouse Dorsal Root Ganglia after Partial Colonic Obstruction. Am. J. Physiol. Gastrointest. Liver Physiol. 289, G670–G678. 10.1152/ajpgi.00028.2005 15920014

[B77] Jarvi K. Laitakari E. M. Koivusalo A. Rintala R. J. Pakarinen M. P. (2010). Bowel Function and Gastrointestinal Quality of Life Among Adults Operated for Hirschsprung Disease during Childhood: a Population-Based Study. Ann. Surg. 252, 977–981. 10.1097/SLA.0b013e3182018542 21107107

[B78] Jia W. Whitehead R. N. Griffiths L. Dawson C. Waring R. H. Ramsden D. B. (2010). Is the Abundance of Faecalibacterium Prausnitzii Relevant to Crohn's Disease? FEMS Microbiol. Lett. 310, 138–144. 10.1111/j.1574-6968.2010.02057.x 20695899 PMC2962807

[B79] Johnson L. A. Rodansky E. S. Haak A. J. Larsen S. D. Neubig R. R. Higgins P. D. (2014). Novel Rho/MRTF/SRF Inhibitors Block Matrix-Stiffness and TGF-β-Induced Fibrogenesis in Human Colonic Myofibroblasts. Inflamm. Bowel Dis. 20, 154–165. 10.1097/01.MIB.0000437615.98881.31 24280883 PMC4893808

[B80] Johnson L. A. Rodansky E. S. Sauder K. L. Horowitz J. C. Mih J. D. Tschumperlin D. J. (2013). Matrix Stiffness Corresponding to Strictured Bowel Induces a Fibrogenic Response in Human Colonic Fibroblasts. Inflamm. Bowel Dis. 19, 891–903. 10.1097/MIB.0b013e3182813297 23502354 PMC3766259

[B81] Kaakoush N. O. Day A. S. Leach S. T. Lemberg D. A. Nielsen S. Mitchell H. M. (2015). Effect of Exclusive Enteral Nutrition on the Microbiota of Children with Newly Diagnosed Crohn's Disease. Clin. Transl Gastroenterol. 6, e71. 10.1038/ctg.2014.21 25588524 PMC4418409

[B82] Kandiel A. Fraser A. G. Korelitz B. I. Brensinger C. Lewis J. D. (2005). Increased Risk of Lymphoma Among Inflammatory Bowel Disease Patients Treated with Azathioprine and 6-mercaptopurine. Gut 54, 1121–1125. 10.1136/gut.2004.049460 16009685 PMC1774897

[B83] Kanefsky J. Lenburg M. Hai C. M. (2006). Cholinergic Receptor and Cyclic Stretch-Mediated Inflammatory Gene Expression in Intact ASM. Am. J. Respir. Cel Mol Biol 34, 417–425. 10.1165/rcmb.2005-0326OC PMC2644203 16339998

[B84] Kansal S. Wagner J. Kirkwood C. D. Catto-Smith A. G. (2013). Enteral Nutrition in Crohn's Disease: an Underused Therapy. Gastroenterol. Res. Pract. 2013, 482108. 10.1155/2013/482108 24382954 PMC3870077

[B85] Katsanos K. H. Tsianos V. E. Maliouki M. Adamidi M. Vagias I. Tsianos E. V. (2010). Obstruction and Pseudo-obstruction in Inflammatory Bowel Disease. Ann. Gastroenterol 23, 243–256.

[B86] Katsumi A. Orr A. W. Tzima E. Schwartz M. A. (2004). Integrins in Mechanotransduction. J. Biol. Chem. 279, 12001–12004. 10.1074/jbc.R300038200 14960578

[B87] Koide A. Yamaguchi T. Odaka T. Koyama H. Tsuyuguchi T. Kitahara H. (2000). Quantitative Analysis of Bowel Gas Using plain Abdominal Radiograph in Patients with Irritable Bowel Syndrome. Am. J. Gastroenterol. 95, 1735–1741. 10.1111/j.1572-0241.2000.02189.x 10925977

[B88] Kraichely R. E. Farrugia G. (2007). Mechanosensitive Ion Channels in Interstitial Cells of Cajal and Smooth Muscle of the Gastrointestinal Tract. Neurogastroenterol Motil. 19, 245–252. 10.1111/j.1365-2982.2006.00880.x 17391240

[B89] Krause W. DuBois R. N. (2000). Eicosanoids and the Large Intestine. Prostaglandins Other Lipid Mediat 61, 145–161. 10.1016/s0090-6980(00)00069-1 10867126

[B90] Kwapiszewska G. Chwalek K. Marsh L. M. Wygrecka M. Wilhelm J. Best J. (2012). BDNF/TrkB Signaling Augments Smooth Muscle Cell Proliferation in Pulmonary Hypertension. Am. J. Pathol. 181, 2018–2029. 10.1016/j.ajpath.2012.08.028 23058367

[B91] Latella G. Rieder F. (2017). Intestinal Fibrosis: Ready to Be Reversed. Curr. Opin. Gastroenterol. 33, 239–245. 10.1097/MOG.0000000000000363 28402994 PMC5572460

[B92] Leach S. T. Mitchell H. M. Eng W. R. Zhang L. Day A. S. (2008). Sustained Modulation of Intestinal Bacteria by Exclusive Enteral Nutrition Used to Treat Children with Crohn's Disease. Aliment. Pharmacol. Ther. 28, 724–733. 10.1111/j.1365-2036.2008.03796.x 19145728

[B93] Levine A. Sigall Boneh R. Wine E. (2018). Evolving Role of Diet in the Pathogenesis and Treatment of Inflammatory Bowel Diseases. Gut 67, 1726–1738. 10.1136/gutjnl-2017-315866 29777041

[B94] Levine A. Wine E. (2013). Effects of Enteral Nutrition on Crohn's Disease: Clues to the Impact of Diet on Disease Pathogenesis. Inflamm. Bowel Dis. 19, 1322–1329. 10.1097/MIB.0b013e3182802acc 23399738

[B95] Levy Nogueira M. da Veiga Moreira J. Baronzio G. F. Dubois B. Steyaert J. M. Schwartz L. (2015). Mechanical Stress as the Common Denominator between Chronic Inflammation, Cancer, and Alzheimer's Disease. Front. Oncol. 5, 197. 10.3389/fonc.2015.00197 26442209 PMC4585184

[B96] Li C. Kuemmerle J. F. (2014). Mechanisms that Mediate the Development of Fibrosis in Patients with Crohn's Disease. Inflamm. Bowel Dis. 20, 1250–1258. 10.1097/MIB.0000000000000043 24831560 PMC4057349

[B97] Li F. Lin Y. M. Sarna S. K. Shi X. Z. (2012a). Cellular Mechanism of Mechanotranscription in Colonic Smooth Muscle Cells. Am. J. Physiol. Gastrointest. Liver Physiol. 303, G646–G656. 10.1152/ajpgi.00440.2011 22700825 PMC3468553

[B98] Li F. Sarna S. K. Shi X. Z. (2012b). Roles of PKCs and PKD in Mechano-Transcription in Colonic Smooth Muscle Cells: Inhibition of Mechano-Transcription as a Potential Treatment for Motility Dysfunction in Obstructive Disorders (Abstract). Gastroenterol 142, S29. 10.1016/s0016-5085(12)60115-2

[B99] Li H. Yin J. Zhang Z. Winston J. H. Shi X. Z. Chen J. D. (2016). Auricular Vagal Nerve Stimulation Ameliorates Burn-Induced Gastric Dysmotility via Sympathetic-COX-2 Pathways in Rats. Neurogastroenterol Motil. 28, 36–42. 10.1111/nmo.12693 26486522 PMC4688125

[B100] Li X. Cao Y. Kan E. M. Lu J. Wong R. K. Shaikh M. (2011). Neuroimmune Regulation of Intestinal Permeability in Inflammatory Bowel Disease (IBD) and Brain-Derived Neurotrophic Factor (BDNF) and Zonulin. Gastroenterol 140 (Suppl. 1), S637–S638. 10.1016/s0016-5085(11)62639-5

[B101] Lin Y. M. Fu Y. Hegde S. Tang Y. Radhakrishnan R. Shi X. Z. (2018). Microsomal Prostaglandin E Synthase-1 Plays a Critical Role in Long-Term Motility Dysfunction after Bowel Obstruction. Sci. Rep. 8, 8831. 10.1038/s41598-018-27230-6 29891860 PMC5995953

[B102] Lin Y. M. Fu Y. Winston J. Radhakrishnan R. Sarna S. K. Huang L. M. (2017). Pathogenesis of Abdominal Pain in Bowel Obstruction: Role of Mechanical Stress-Induced Upregulation of Nerve Growth Factor in Gut Smooth Muscle Cells. Pain 158, 583–592. 10.1097/j.pain.0000000000000797 28079757 PMC5354958

[B103] Lin Y. M. Fu Y. Wu C. C. Xu G. Y. Huang L. Y. Shi X. Z. (2015). Colon Distention Induces Persistent Visceral Hypersensitivity by Mechanotranscription of Pain Mediators in Colonic Smooth Muscle Cells. Am. J. Physiol. Gastrointest. Liver Physiol. 308, G434–G441. 10.1152/ajpgi.00328.2014 25540231 PMC4346753

[B104] Lin Y. M. Li F. Choudhury B. Winston J. H. Sarna S. K. Shi X. Z. (2014a). Effects of Mechanical Stress on Myenteric Neurons in the colon. Gastroenterol 146 (Suppl. 1), S91. 10.1016/s0016-5085(14)60330-9

[B105] Lin Y. M. Li F. Shi X. Z. (2014b). Mechanical Stress Is a Pro-inflammatory Stimulus in the Gut: *In Vitro*, *In Vivo* and *Ex Vivo* Evidence. PLoS One 9, e106242. 10.1371/journal.pone.0106242 25180799 PMC4152012

[B106] Lin Y. M. Li F. Shi X. Z. (2012a). Mechano-transcription of COX-2 Is a Common Response to Lumen Dilation of the Rat Gastrointestinal Tract. Neurogastroenterol Motil. 24, 670–676. 10.1111/j.1365-2982.2012.01918.x 22489918 PMC4183192

[B107] Lin Y. M. Qiu S. M'Koma A. E. Powell D. W. Cohn S. Shi X. Z. (2021a). Mechanical Stress Plays a Critical Role in Intestinal Fibrosis and Smooth Muscle Hyperplasia in a Rodent Model of Crohn’s Disease (Abstract). Gastroenterol 160 (Suppl. 1), S431. 10.1016/s0016-5085(21)01743-1

[B108] Lin Y. M. Sarna S. K. Shi X. Z. (2012b). Prophylactic and Therapeutic Benefits of COX-2 Inhibitor on Motility Dysfunction in Bowel Obstruction: Roles of PGE₂ and EP Receptors. Am. J. Physiol. Gastrointest. Liver Physiol. 302, G267–G275. 10.1152/ajpgi.00326.2011 22038825 PMC3341114

[B109] Lin Y. M. Shi X. Z. (2021). Preclinical Studies of Pathophysiological Role and Diagnostic Potential of Osteopontin in Crohn’s Disease (Abstract). Gastroenterol 160 (Suppl. 1), S626–S627. 10.1016/s0016-5085(21)02202-2

[B110] Lin Y. M. Tang Y. Fu Y. Hegde S. Shi D. W. Huang L. M. (2021b). An Opioid Receptor-independent Mechanism Underlies Motility Dysfunction and Visceral Hyperalgesia in Opioid-Induced Bowel Dysfunction. Am. J. Physiol. Gastrointest. Liver Physiol. 320, G1093–G1104. 10.1152/ajpgi.00400.2020 33908261 PMC8285582

[B111] Linan-Rico A. Ochoa-Cortes F. Beyder A. Soghomonyan S. Zuleta-Alarcon A. Coppola V. (2016). Mechanosensory Signaling in Enterochromaffin Cells and 5-HT Release: Potential Implications for Gut Inflammation. Front. Neurosci. 10, 564. 10.3389/fnins.2016.00564 28066160 PMC5165017

[B112] Liu L. Shang F. Morgan M. J. King D. W. Lubowski D. Z. Burcher E. (2009). Cyclooxygenase-dependent Alterations in Substance P-Mediated Contractility and Tachykinin NK1 Receptor Expression in the Colonic Circular Muscle of Patients with Slow Transit Constipation. J. Pharmacol. Exp. Ther. 329, 282–289. 10.1124/jpet.108.148148 19164461

[B113] Lovell R. M. Ford A. C. (2012). Global Prevalence of and Risk Factors for Irritable Bowel Syndrome: a Meta-Analysis. Clin. Gastroenterol. Hepatol. 10, 712–e4. 10.1016/j.cgh.2012.02.029 22426087

[B114] Lu C. L. Pasricha P. J. Hsieh J. C. Lu R. H. Lai C. R. Wu L. L. (2005). Changes of the Neuropeptides Content and Gene Expression in Spinal Cord and Dorsal Root Ganglion after Noxious Colorectal Distension. Regul. Pept. 131, 66–73. 10.1016/j.regpep.2005.06.008 16084604

[B115] MacLellan A. Moore-Connors J. Grant S. Cahill L. Langille M. G. I. Van Limbergen J. (2017). The Impact of Exclusive Enteral Nutrition (EEN) on the Gut Microbiome in Crohn's Disease: a Review. Nutrients 9, 447. 10.3390/nu9050447 PMC5452177 28468301

[B116] Mayer E. A. Gebhart G. F. (1994). Basic and Clinical Aspects of Visceral Hyperalgesia. Gastroenterology 107, 271–293. 10.1016/0016-5085(94)90086-8 8020671

[B117] Menezes M. Corbally M. Puri P. (2006). Long-term Results of Bowel Function after Treatment for Hirschsprung's Disease: a 29-year Review. Pediatr. Surg. Int. 22, 987–990. 10.1007/s00383-006-1783-8 17006709

[B118] Million M. Wang L. Wang Y. Adelson D. W. Yuan P. Q. Maillot C. (2006). CRF2 Receptor Activation Prevents Colorectal Distension Induced Visceral Pain and Spinal ERK1/2 Phosphorylation in Rats. Gut. 55, 172–181. 10.1136/gut.2004.051391 15985561 PMC1856510

[B119] Mohajer B. Ma T. Y. (2000). Eicosanoids and the Small Intestine. Prostaglandins Other Lipid Mediat 61, 125–143. 10.1016/s0090-6980(00)00068-x 10867125

[B120] Mowat C. Cole A. Windsor A. Ahmad T. Arnott I. Driscoll R. IBD Section of the British Society of Gastroenterology (2011). Guidelines for the Management of Inflammatory Bowel Disease in Adults. Gut. 60, 571–607. 10.1136/gut.2010.224154 21464096

[B121] Munakata J. Naliboff B. Harraf F. Kodner A. Lembo T. Chang L. (1997). Repetitive Sigmoid Stimulation Induces Rectal Hyperalgesia in Patients with Irritable Bowel Syndrome. Gastroenterology 112, 55–63. 10.1016/s0016-5085(97)70219-1 8978343

[B122] Murthy K. S. (2006). Signaling for Contraction and Relaxation in Smooth Muscle of the Gut. Annu. Rev. Physiol. 68, 345–374. 10.1146/annurev.physiol.68.040504.094707 16460276

[B123] Ness T. J. Metcalf A. M. Gebhart G. F. (1990). A Psychophysiological Study in Humans Using Phasic Colonic Distension as a Noxious Visceral Stimulus. Pain 43, 377–386. 10.1016/0304-3959(90)90035-c 2293146

[B124] Nunez R. Blesa E. Cabrera R. (2009). “Hirschsprung’s Disease: Clinical Features,” in Hirschsprung’s Disease: Diagnosis and Treatment. Editors Nunez R. N. Lopez-Alonso M. (New York: Nova Science Publishers), 125–136.

[B125] O'Moráin C. Segal A. W. Levi A. J. (1984). Elemental Diet as Primary Treatment of Acute Crohn's Disease: a Controlled Trial. Br. Med. J. (Clin Res. Ed. 288, 1859–1862. 10.1136/bmj.288.6434.1859 PMC1441790 6428577

[B126] Olaison G. Smedh K. Sjödahl R. (1992). Natural Course of Crohn's Disease after Ileocolic Resection: Endoscopically Visualised Ileal Ulcers Preceding Symptoms. Gut 33, 331–335. 10.1136/gut.33.3.331 1568651 PMC1373822

[B127] Papadakis K. A. Targan S. R. (2000). Role of Cytokines in the Pathogenesis of Inflammatory Bowel Disease. Annu. Rev. Med. 51, 289–298. 10.1146/annurev.med.51.1.289 10774465

[B128] Pezet S. McMahon S. B. (2006). Neurotrophins: Mediators and Modulators of Pain. Annu. Rev. Neurosci. 29, 507–538. 10.1146/annurev.neuro.29.051605.112929 16776595

[B129] Prihoda M. Flatt A. Summers R. W. (1984). Mechanisms of Motility Changes during Acute Intestinal Obstruction in the Dog. Am. J. Physiol. 247, G37–G42. 10.1152/ajpgi.1984.247.1.G37 6742196

[B130] Quince C. Ijaz U. Z. Loman N. Eren A. M. Saulnier D. Russell J. (2015). Extensive Modulation of the Fecal Metagenome in Children with Crohn's Disease during Exclusive Enteral Nutrition. Am. J. Gastroenterol. 110, 1718–1730. 10.1038/ajg.2015.357 26526081 PMC4697132

[B131] Raahave D. (2015). Faecal Retention: a Common Cause in Functional Bowel Disorders, Appendicitis and Haemorrhoids-Wwith Medical and Surgical Therapy. Dan Med. J. 62, B5031. 25748875

[B132] Raahave D. Jensen A. K. (2021). Increased colon Transit Time and Faecal Load in Irritable Bowel Syndrome. World J. Gastrointest. Pharmacol. Ther. 12, 13–20. 10.4292/wjgpt.v12.i1.13 33564493 PMC7844574

[B133] Raju D. P. Hakendorf P. Costa M. Wattchow D. A. (2015). Efficacy and Safety of Low-Dose Celecoxib in Reducing post-operative Paralytic Ileus after Major Abdominal Surgery. ANZ J. Surg. 85, 946–950. 10.1111/ans.12475 26780018

[B134] Rieder F. Fiocchi C. Rogler G. (2017). Mechanisms, Management, and Treatment of Fibrosis in Patients with Inflammatory Bowel Diseases. Gastroenterology 152, 340–e6. 10.1053/j.gastro.2016.09.047 27720839 PMC5209279

[B135] Rieder F. Zimmermann E. M. Remzi F. H. Sandborn W. J. (2013). Crohn's Disease Complicated by Strictures: a Systematic Review. Gut 62, 1072–1084. 10.1136/gutjnl-2012-304353 23626373 PMC4884453

[B136] Ripamonti C. Mercadante S. (2004). “Pathophysiology and Management of Malignant Bowel Obstruction,” in Oxford Textbook of Palliative Medicine. Editors Doyle D. Hanks G. W. C. Cherny N. Calman K. (New York, NY: Oxford University Press), 3, 496–507.

[B137] Ripamonti C. Twycross R. Baines M. Bozzetti F. Capri S. De Conno F. (2001). Clinical-practice Recommendations for the Management of Bowel Obstruction in Patients with End-Stage Cancer. Support Care Cancer 9, 223–233. 10.1007/s005200000198 11430417

[B138] Rittling S. R. Singh R. (2015). Osteopontin in Immune-Mediated Diseases. J. Dent Res. 94, 1638–1645. 10.1177/0022034515605270 26341976 PMC4681477

[B139] Roeland E. von Gunten C. F. (2009). Current Concepts in Malignant Bowel Obstruction Management. Curr. Oncol. Rep. 11, 298–303. 10.1007/s11912-009-0042-2 19508835

[B140] Ruemmele F. M. Lachaux A. Cézard J. P. Morali A. Maurage C. Giniès J. L. (2009). Efficacy of Infliximab in Pediatric Crohn's Disease: a Randomized Multicenter Open-Label Trial Comparing Scheduled to on Demand Maintenance Therapy. Inflamm. Bowel Dis. 15, 388–394. 10.1002/ibd.20788 19023899

[B141] Ruemmele F. M. Veres G. Kolho K. L. Griffiths A. Levine A. Escher J. C. (2014). European Crohn's and Colitis Organisation; European Society of Pediatric Gastroenterology, Hepatology and NutritionConsensus Guidelines of ECCO/ESPGHAN on the Medical Management of Pediatric Crohn's Disease. J. Crohns Colitis 8, 1179–1207. 10.1016/j.crohns.2014.04.005 24909831

[B142] Russell J. C. Welch J. P. (1990). “Pathophysiology of Bowel Obstruction,” in Bowel Obstruction. Editor Welch J. P. (Philadelphia, PA: W.B. Saunders), 28–58.

[B143] Rutgeerts P. Goboes K. Peeters M. Hiele M. Penninckx F. Aerts R. (1991). Effect of Faecal Stream Diversion on Recurrence of Crohn's Disease in the Neoterminal Ileum. Lancet 338, 771–774. 10.1016/0140-6736(91)90663-a 1681159

[B144] Ruwhof C. van der Laarse A. (2000). Mechanical Stress-Induced Cardiac Hypertrophy: Mechanisms and Signal Transduction Pathways. Cardiovasc. Res. 47, 23–37. 10.1016/s0008-6363(00)00076-6 10869527

[B145] Sanderson I. R. Boulton P. Menzies I. Walker-Smith J. A. (1987). Improvement of Abnormal Lactulose/rhamnose Permeability in Active Crohn's Disease of the Small Bowel by an Elemental Diet. Gut. 28, 1073–1076. 10.1136/gut.28.9.1073 3678965 PMC1433221

[B146] Saps M. Bonilla S. (2011). Early Life Events: Infants with Pyloric Stenosis Have a Higher Risk of Developing Chronic Abdominal Pain in Childhood. J. Pediatr. 159, 551–e1. 10.1016/j.jpeds.2011.03.018 21513946

[B147] Sarna S. K. Shi X.-Z. (2006). “Function and Regulation of Colonic Contractions in Health and Disease,” in Physiology of the Gastrointestinal Tract. Editor Johnson L. R. (Amsterdam: Elsevier Academic Press), 1, 965–993. 10.1016/b978-012088394-3/50042-8

[B148] Sartor R. B. (2006). Mechanisms of Disease: Pathogenesis of Crohn's Disease and Ulcerative Colitis. Nat. Clin. Pract. Gastroenterol. Hepatol. 3, 390–407. 10.1038/ncpgasthep0528 16819502

[B149] Sato T. Nakai T. Tamura N. Okamoto S. Matsuoka K. Sakuraba A. (2005). Osteopontin/Eta-1 Upregulated in Crohn's Disease Regulates the Th1 Immune Response. Gut 54, 1254–1262. 10.1136/gut.2004.048298 16099792 PMC1774642

[B150] Satsangi J. Silverberg M. S. Vermeire S. Colombel J. F. (2006). The Montreal Classification of Inflammatory Bowel Disease: Controversies, Consensus, and Implications. Gut 55, 749–753. 10.1136/gut.2005.082909 16698746 PMC1856208

[B151] Serra J. Azpiroz F. Malagelada J. R. (2001). Impaired Transit and Tolerance of Intestinal Gas in the Irritable Bowel Syndrome. Gut 48, 14–19. 10.1136/gut.48.1.14 11115817 PMC1728167

[B152] Sharma A. Rao S. (2017). Constipation: Pathophysiology and Current Therapeutic Approaches. Handb Exp. Pharmacol. 239, 59–74. 10.1007/164_2016_111 28185025

[B153] Shi X. Z. Lin Y. M. Hegde S. (2018). Novel Insights into the Mechanisms of Abdominal Pain in Obstructive Bowel Disorders. Front. Integr. Neurosci. 12, 23. 10.3389/fnint.2018.00023 29937720 PMC6002527

[B154] Shi X. Z. Lin Y. M. Powell D. W. Sarna S. K. (2011). Pathophysiology of Motility Dysfunction in Bowel Obstruction: Role of Stretch-Induced COX-2. Am. J. Physiol. Gastrointest. Liver Physiol. 300, G99–G108. 10.1152/ajpgi.00379.2010 21051526 PMC3025501

[B155] Shi X. Z. (2017). Mechanical Regulation of Gene Expression in Gut Smooth Muscle Cells. Front. Physiol. 8, 1000. 10.3389/fphys.2017.01000 29259559 PMC5723328

[B156] Shi X. Z. Sarna S. K. (2005). Transcriptional Regulation of Inflammatory Mediators Secreted by Human Colonic Circular Smooth Muscle Cells. Am. J. Physiol. Gastrointest. Liver Physiol. 289, G274–G284. 10.1152/ajpgi.00512.2004 15790759

[B157] Shikata J. Shida T. Amino K. Ishioka K. (1983). Experimental Studies on the Hemodynamics of the Small Intestine Following Increased Intraluminal Pressure. Surg. Gynecol. Obstet. 156, 155–160. 6823651

[B158] Shim L. Prott G. Hansen R. D. Simmons L. E. Kellow J. E. Malcolm A. (2010). Prolonged Balloon Expulsion Is Predictive of Abdominal Distension in Bloating. Am. J. Gastroenterol. 105, 883–887. 10.1038/ajg.2010.54 20179695

[B159] Sidoroff M. Kolho K. L. (2012). Glucocorticoids in Pediatric Inflammatory Bowel Disease. Scand. J. Gastroenterol. 47, 745–750. 10.3109/00365521.2012.679681 22507033

[B160] Silen W. (2005). “Acute Intestinal Obstruction,” in Harrison's Principles of Internal Medicine. Editors Kasper D. L. Braunwald E. Hauser S. Longo D. Jameson J. L. Fauci A. S.. 16th ed. (New York, NY: McGraw-Hill), 2, 1803–1805.

[B161] Slugg R. M. Meyer R. A. Campbell J. N. (2000). Response of Cutaneous A- and C-Fiber Nociceptors in the Monkey to Controlled-Force Stimuli. J. Neurophysiol. 83, 2179–2191. 10.1152/jn.2000.83.4.2179 10758127

[B162] Sokol H. Pigneur B. Watterlot L. Lakhdari O. Bermúdez-Humarán L. G. Gratadoux J. J. (2008). Faecalibacterium Prausnitzii Is an Anti-inflammatory Commensal Bacterium Identified by Gut Microbiota Analysis of Crohn Disease Patients. Proc. Natl. Acad. Sci. U S A. 105, 16731–16736. 10.1073/pnas.0804812105 18936492 PMC2575488

[B163] Stadnyk A. W. (2002). Intestinal Epithelial Cells as a Source of Inflammatory Cytokines and Chemokines. Can. J. Gastroenterol. 16, 241–246. 10.1155/2002/941087 11981577

[B164] Steinkamp M. Schulte N. Spaniol U. Pflüger C. Hartmann C. Kirsch J. (2012). Brain Derived Neurotrophic Factor Inhibits Apoptosis in Enteric Glia during Gut Inflammation. Med. Sci. Monit. 18, BR117–22. 10.12659/msm.882612 22460084 PMC3560818

[B165] Steinsvik E. K. Sangnes D. A. Søfteland E. Biermann M. Assmus J. Dimcevski G. (2021). Gastric Function in Diabetic Gastroparesis Assessed by Ultrasound and Scintigraphy. Neurogastroenterology Motil. 1, e14235. 10.1111/nmo.14235 34378839

[B166] Suchyna T. M. Johnson J. H. Hamer K. Leykam J. F. Gage D. A. Clemo H. F. (2000). Identification of a Peptide Toxin from Grammostola Spatulata Spider Venom that Blocks Cation-Selective Stretch-Activated Channels. J. Gen. Physiol. 115, 583–598. 10.1085/jgp.115.5.583 10779316 PMC2217226

[B167] Summers R. W. Yanda R. Prihoda M. Flatt A. (1983). Acute Intestinal Obstruction: an Electromyographic Study in Dogs. Gastroenterology 85, 1301–1306. 10.1016/s0016-5085(83)80010-9 6628927

[B168] Summers R. W. (1999). Chapter 39, Approach to the Patient with Ileus and Obstruction. In Ed, Yamada T. Alpers D. H. Laine L. Owyang C. Powell D. W. (Eds.), Textbook of Gastroenterology. 1, Lippincott Williams & Wilkins, Philadelphia, PA, pp. 842- 858.

[B169] Sun W. M. Doran S. M. Jones K. L. Davidson G. Dent J. Horowitz M. (2000). Long-term Effects of Pyloromyotomy on Pyloric Motility and Gastric Emptying in Humans. Am. J. Gastroenterol. 95, 92–100. 10.1111/j.1572-0241.2000.01705.x 10638565

[B170] Swaminath A. Feathers A. Ananthakrishnan A. N. Falzon L. Li Ferry S. (2017). Systematic Review with Meta-Analysis: Enteral Nutrition Therapy for the Induction of Remission in Paediatric Crohn's Disease. Aliment. Pharmacol. Ther. 46, 645–656. 10.1111/apt.14253 28815649 PMC5798240

[B171] Sweetser S. Rao A. S. Szarka L. A. (2012). Constipation and Recurrent Abdominal Distension in a 39-Year-Old Woman with Irritable Bowel Syndrome. Gut 61 (42), 42–107. 10.1136/gutjnl-2011-300925 21890813

[B172] Terry N. Margolis K. G. (2017). Serotonergic Mechanisms Regulating the GI Tract: Experimental Evidence and Therapeutic Relevance. Handb Exp. Pharmacol. 239, 319–342. 10.1007/164_2016_103 28035530 PMC5526216

[B173] Thompson J. S. (2006). Overview of Etiology and Management of Intestinal Failure. Gastroenterology 130 (Suppl. 1), S3–S4. 10.1053/j.gastro.2005.09.062 16473069

[B174] Tschumperlin D. J. Ligresti G. Hilscher M. B. Shah V. H. (2018). Mechanosensing and Fibrosis. J. Clin. Invest. 128, 74–84. 10.1172/JCI93561 29293092 PMC5749510

[B175] Uede T. (2011). Osteopontin, Intrinsic Tissue Regulator of Intractable Inflammatory Diseases. Pathol. Int. 61, 265–280. 10.1111/j.1440-1827.2011.02649.x 21501293

[B176] Verma S. Brown S. Kirkwood B. Giaffer M. H. (2000). Polymeric versus Elemental Diet as Primary Treatment in Active Crohn's Disease: a Randomized, Double-Blind Trial. Am. J. Gastroenterol. 95, 735–739. 10.1111/j.1572-0241.2000.01527.x 10710067

[B177] Voitk A. J. Echave V. Feller J. H. Brown R. A. Gurd F. N. (1973). Experience with Elemental Diet in the Treatment of Inflammatory Bowel Disease. Is This Primary Therapy? Arch. Surg. 107, 329–333. 10.1001/archsurg.1973.01350200189039 4198183

[B178] Wattchow D. A. De Fontgalland D. Bampton P. A. Leach P. L. McLaughlin K. Costa M. (2009). Clinical Trial: the Impact of Cyclooxygenase Inhibitors on Gastrointestinal Recovery after Major Surgery - a Randomized Double Blind Controlled Trial of Celecoxib or Diclofenac vs. Placebo. Aliment. Pharmacol. Ther. 30, 987–998. 10.1111/j.1365-2036.2009.04126.x 19694636

[B179] Wedel T. Spiegler J. Soellner S. Roblick U. J. Schiedeck T. H. Bruch H. P. (2002). Enteric Nerves and Interstitial Cells of Cajal Are Altered in Patients with Slow-Transit Constipation and Megacolon. Gastroenterology 123, 1459–1467. 10.1053/gast.2002.36600 12404220

[B180] Wehner S. Buchholz B. M. Schuchtrup S. Rocke A. Schaefer N. Lysson M. (2010). Mechanical Strain and TLR4 Synergistically Induce Cell-specific Inflammatory Gene Expression in Intestinal Smooth Muscle Cells and Peritoneal Macrophages. Am. J. Physiol. Gastrointest. Liver Physiol. 299, G1187–G1197. 10.1152/ajpgi.00452.2009 20829523

[B181] West S. D. Suliburk J. W. Smith G. S. Mercer D. W. (2006). Effects of Lipopolysaccharide on Gastric Stasis: Role of Cyclooxygenase. Dig. Dis. Sci. 51, 754–765. 10.1007/s10620-006-3203-2 16615000

[B182] Williams S. B. Greenspon J. Young H. A. Orkin B. A. (2005). Small Bowel Obstruction: Conservative vs. Surgical Management. Dis. Colon Rectum 48, 1140–1146. 10.1007/s10350-004-0882-7 15906139

[B183] Winston J. Toma H. Shenoy M. Pasricha P. J. (2001). Nerve Growth Factor Regulates VR-1 mRNA Levels in Cultures of Adult Dorsal Root Ganglion Neurons. Pain 89, 181–186. 10.1016/s0304-3959(00)00370-5 11166474

[B184] Won K. J. Suzuki T. Hori M. Ozaki H. (2006). Motility Disorder in Experimentally Obstructed Intestine: Relationship between Muscularis Inflammation and Disruption of the ICC Network. Neurogastroenterol Motil. 18, 53–61. 10.1111/j.1365-2982.2005.00718.x 16371083

[B185] Wu C. C. Lin Y. M. Gao J. Winston J. H. Cheng L. K. Shi X. Z. (2013). Are Interstitial Cells of Cajal Involved in Mechanical Stress-Induced Gene Expression and Impairment of Smooth Muscle Contractility in Bowel Obstruction? PLoS One 8, e76222. 10.1371/journal.pone.0076222 24098782 PMC3786942

[B186] Yamaguchi O. (2004). Response of Bladder Smooth Muscle Cells to Obstruction: Signal Transduction and the Role of Mechanosensors. Urology 63 (Suppl. 1), 11–16. 10.1016/j.urology.2003.12.002 15013647

[B187] Yamamoto T. Fazio V. W. Tekkis P. P. (2007). Safety and Efficacy of Strictureplasty for Crohn's Disease: A Systematic Review and Meta-Analysis. Dis. Colon Rectum 50, 1968–1986. 10.1007/s10350-007-0279-5 17762967

[B188] Yang Q. Gao X. Chen H. Li M. Wu X. Zhi M. (2017). Efficacy of Exclusive Enteral Nutrition in Complicated Crohn's Disease. Scand. J. Gastroenterol. 52, 995–1001. 10.1080/00365521.2017.1335770 28598298

[B189] Yu Y. B. Zuo X. L. Zhao Q. J. Chen F. X. Yang J. Dong Y. Y. (2012). Brain-derived Neurotrophic Factor Contributes to Abdominal Pain in Irritable Bowel Syndrome. Gut 61, 685–694. 10.1136/gutjnl-2011-300265 21997550

[B190] Zachos M. Tondeur M. Griffiths A. M. (2007). Enteral Nutritional Therapy for Induction of Remission in Crohn's Disease. Cochrane Database Syst. Rev. 1, CD000542. 10.1002/14651858.CD000542.pub2 17253452

[B191] Zar S. Benson M. J. Kumar D. (2002). Review Article: Bloating in Functional Bowel Disorders. Aliment. Pharmacol. Ther. 16, 1867–1876. 10.1046/j.1365-2036.2002.01369.x 12390095

[B192] Ziegler A. L. Blikslager A. T. (2020). Sparing the Gut: COX-2 Inhibitors herald a new era for Treatment of Horses with Surgical Colic. Equine Vet. Educ. 32, 611–616. 10.1111/eve.13189 34305336 PMC8297937

[B193] Ziegler A. L. Freeman C. K. Fogle C. A. Burke M. J. Davis J. L. Cook V. L. (2019). Multicentre, Blinded, Randomised Clinical Trial Comparing the Use of Flunixin Meglumine with Firocoxib in Horses with Small Intestinal Strangulating Obstruction. Equine Vet. J. 51, 329–335. 10.1111/evj.13013 30156312 PMC6788450

